# Dynamic remodeling of amino acid metabolism in tumor-associated macrophages: Fueling immunosuppression, reshaping tumor niches, and unlocking metabolic checkpoints

**DOI:** 10.1016/j.jare.2025.09.025

**Published:** 2025-09-18

**Authors:** Beibei Ran, Lingjun Xiao, Yan Liu, Chenglin Zhang, Lingkai Kong, Yuxin Zhang, Xiaosong Gu, Chunping Jiang, Junhua Wu

**Affiliations:** a State Key Laboratory of Pharmaceutical Biotechnology, Department of General Surgery Nanjing Drum Tower Hospital, The Affiliated Hospital of Medical School, Medical School, Nanjing University, Nanjing 210008, China; b Jinan Microecological Biomedicine Shandong Laboratory, Jinan 250021, China; c “Nanjing University-Gulou” Joint Laboratory of AI and Healthcare BigData, National Institute of Healthcare Data Science at Nanjing University, School of Life Sciences, Jiangsu Key Laboratory of Molecular Medicine, Nanjing University, Nanjing 210093, China; d Department of Hepatobiliary and Pancreatic Surgery, The Second Affiliated Hospital of Fujian Medical University, 362000 Quanzhou, Fujian Province, China; e Renhuai People's Hospital, 564055 Renhuai, Guizhou Province, China

**Keywords:** Tumor-associated macrophages (TAMs), Amino acid, Metabolic reprogramming, Tumor microenvironment (TME), Metabolic crosstalk, Immunometabolic crosstalk, Immunotherapy, Therapeutic target, M1-like TAMs, M2-like TAMs

## Abstract

**Background:**

Tumor-associated macrophages (TAMs) depend on their amino acid metabolism to determine their properties and immune function and play important roles in the tumor microenvironment (TME). Although in previous studies, targeting amino acid metabolism to transform the protumor function of TAMs into antitumor immune function has shown promising application as a tumor therapy, current clinical research is still limited. There is a lack of discussion on the mechanism and treatment strategy for determining tumor progression by controlling amino acid metabolism in TAMs, as does a summary of studies on promoting tumor progression by reshaping amino acid metabolism in TAMs.

**Aim of review:**

This review aims to systematically review and summarize the crosstalk between amino acid metabolism in TAMs and the TME, analyze the determining role of its metabolic network in tumor occurrence and development, and summarize therapies on this basis to help determine the development status and emerging technologies in the field of amino acid metabolism in TAMs for tumor therapy.

**Key scientific concepts of review:**

This review dissects how TAMs exploit amino acid dynamics via transporters, enzymes, and sensors to adopt protumoral phenotypes, depleting critical metabolites and crippling antitumor T-cell responses. We map the immunometabolic crosstalk through which TAMs reshape immunity, highlighting nutrient competition and metabolic byproducts as dual drivers of immune dysfunction. Emerging therapeutic strategies targeting these pathways (IFN-γ-JAK-STAT1 and IL-6/JAK2/STAT3) have been critically evaluated for their potential to reprogram TAMs and reverse immunosuppression. Key challenges, such as TAM heterogeneity, metabolic plasticity, and therapy resistance, are addressed, emphasizing the need for single-cell-resolution mapping of TAM metabolic states to identify context-dependent vulnerabilities. Finally, we advocate for combinatorial approaches that couple metabolic rewiring with immunotherapies, proposing that disrupting amino acid dependencies in TAMs could dismantle the immunosuppressive TME.

## Highlights

• A new perspective on amino acid metabolism in TAMs in tumor microenvironment.
• Novel strategies for amino acid metabolism remodeling in TAMs.
• A summary concerning amino acid metabolism in TAMs therapies and tumor therapy.
• The deficiency of amino acid metabolism in TAMs and future development and clinical application were proposed.

## Graphical abstract

Considering the impact of amino acid metabolism in TAMs on tumor therapy, this review summarizes and analyzes the immunometabolic features of amino acid in tumor-associated macrophages (TAMs) and its crosstalk with various aspects of tumor microenvironment (TME), and investigates the potential as a biomarker of immune response, indicates combination of immunotherapy and metabolic intervention could better unlock the potential of anti-cancer therapies.

**Figure:**
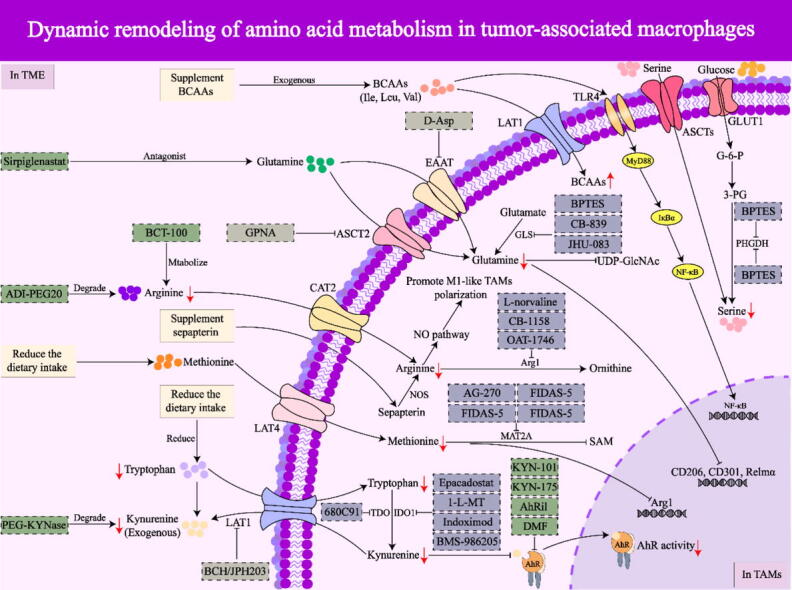

## Introduction

**Fig. 1:**
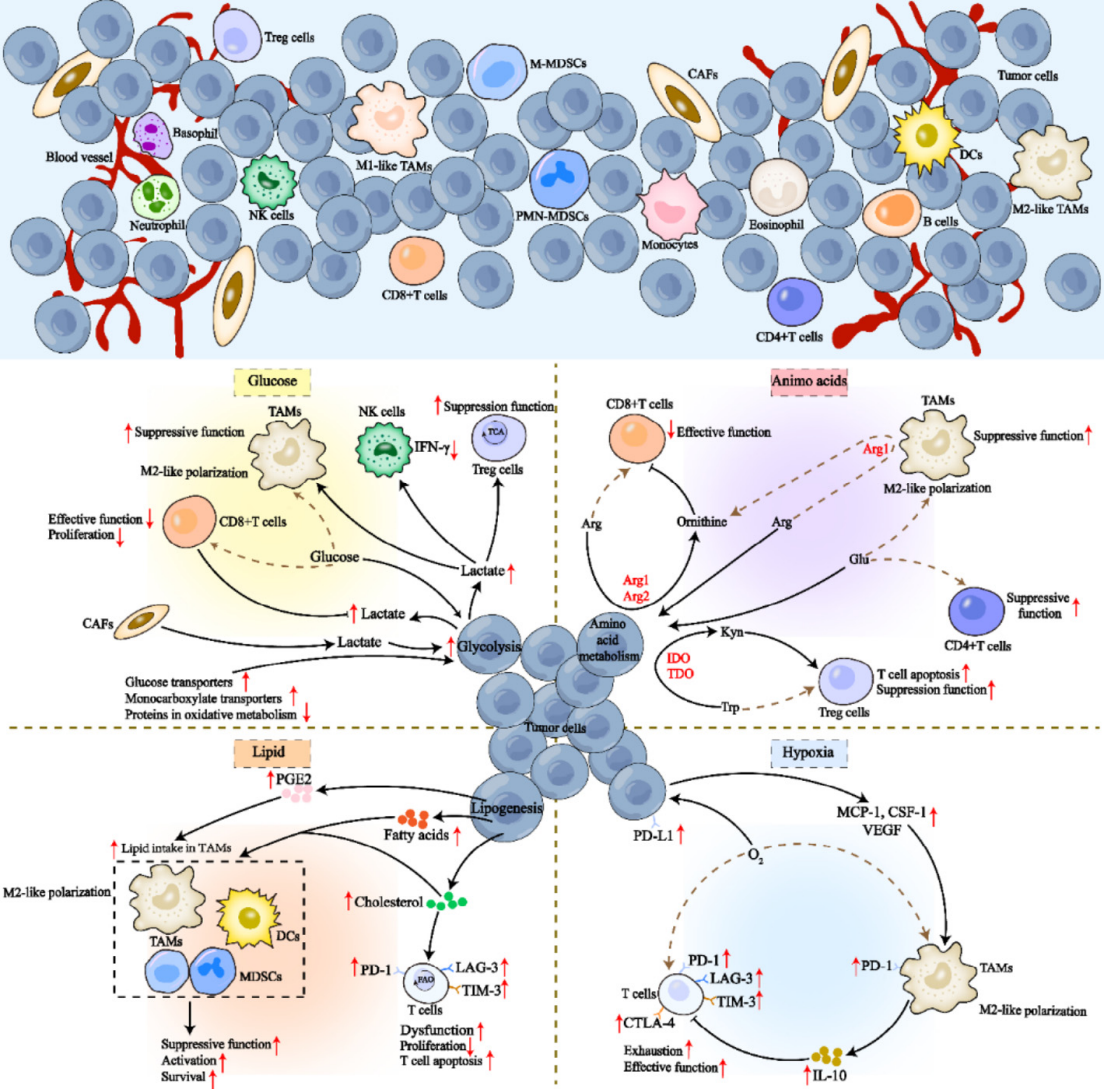
Alterations in immune cell metabolism and functions in the TME. *Arg*, arginine; *Arg1/2*, arginase 1/2; *CAFs*, cancer-associated fibroblasts; *CSF-1*, macrophage colony stimulating factor-1; *CTLA 4*, cytotoxic T lymphocyte associate protein 4; *DCs*, dendritic cells; *Glu*, glutamine; *IDO1*, indoleamine2,3-dioxygenase1; *IL-10*, interleukin 10; *INF-γ*, interferon-γ; *Kyn*, kynurenine; *LAG-3*, lymphocyte activation gene-3; *MCP-1*, monocyte chemotactic protein-1; *M-MDSCs*, monocytic-myeloid-derived suppressor cells; *PD-1*, programmed death 1; *PD-L1*, programmed death ligand-1; *PGE2*, prostaglandin E2; *PMN-MDSCs*, polymorphonuclear-myeloid-derived suppressor cells; *TAMs*, tumor-associated macrophages; *TDO*, tryptophan-2,3-dioxygenase; *TIM-3*, T-cell immunoglobulin domain and mucin domain-3; *Trp*, tryptophan; *VEGF*, vascular endothelial growth factor.

Metabolic reprogramming (the process by which cells adjust their energy and material metabolism to meet the demands of physiological and pathological conditions) has emerged as a hallmark of cancer, as the unique metabolic pathway has led to reshaping of the tumor microenvironment (TME) and reversing a promising strategy for tumor immune escape [[1], [2], [3], [4], [5], [6]]. To adapt to hypoxia, stress, unrestricted proliferation, and nutrient deficiency microenvironments, tumor cells are compelled to increase lipid metabolism and glucose uptake and undergo amino acid metabolism reprogramming to fulfill their own demands [[7], [8], [9]]. Abnormal amino acid metabolism in tumor cells, such as increased uptake and changes in metabolic pathways, products, or key enzymes [10], can lead to a deficiency in amino acids or the accumulation of specific products in the TME, inhibiting antitumor immunity [11,12]. As the main immune cell population in tumors, tumor-associated macrophages (TAMs) also rely on amino acids for functional adaptation to the TME (Fig. 1).

To accommodate metabolic and signal alterations in the TME, TAMs can stringently cause metabolic reprogramming and obtain immunosuppressive (the state of the immune system in the tumor microenvironment is not functioning well) and tumor-promoting functions [13,14]. In the TME, TAMs highly utilize amino acid metabolism to promote their tumorigenic state and functional transformation [15,16]. After polarization, TAMs possess a significant capacity to take up and metabolize tryptophan and arginine and mainly exhibit the phenotypic and functional characteristics of immunosuppressive macrophages [17,18]. In addition, amino acid metabolism in TAMs can trigger the exchange of substances and signals of cytokines, metabolites and signaling molecules with other cells, reshaping the TME state [[19], [20], [21], [22]]. Targeting amino acid metabolism to transform the tumor-promoting functions of TAMs into antitumor immunity represents a new strategy for tumor immunotherapy and has important application prospects. Therefore, how TAMs acquire tumor-promoting functions by reshaping amino acid metabolism and how the immunoregulatory network of TAMs affects tumor progression and response to therapy are currently important topics in the field of tumor immunology.

Robust biological principles and prospective biomarkers must be integrated when evaluating drugs that target amino acid metabolism in TAMs. At present, only a limited number of amino acid and regulatory pathways have been examined, with few in-depth analyses of the immunometabolic features of amino acid metabolism and its potential as an immune-targeting marker. In this review, particular emphasis is placed on the interplay between various factors involved in amino acid metabolic reprogramming in TAMs and their implications for therapeutic targets and biomarkers in tumors. Additionally, this review examines how amino acid metabolic reprogramming in TAMs contributes to protumor effects and summarizes and categorizes the latest strategies from clinical trials aimed at reversing the amino acid immunometabolic features of TAMs, dividing these approaches into three categories: direct deprivation of amino acids in the external environment, targeting amino acid membrane transporters, and modulating key enzymes and sensors in metabolic pathways.

## Recruitment, phenotypes and metabolic profiles of TAMs

### Origin and recruitment of TAMs

**Fig. 2:**
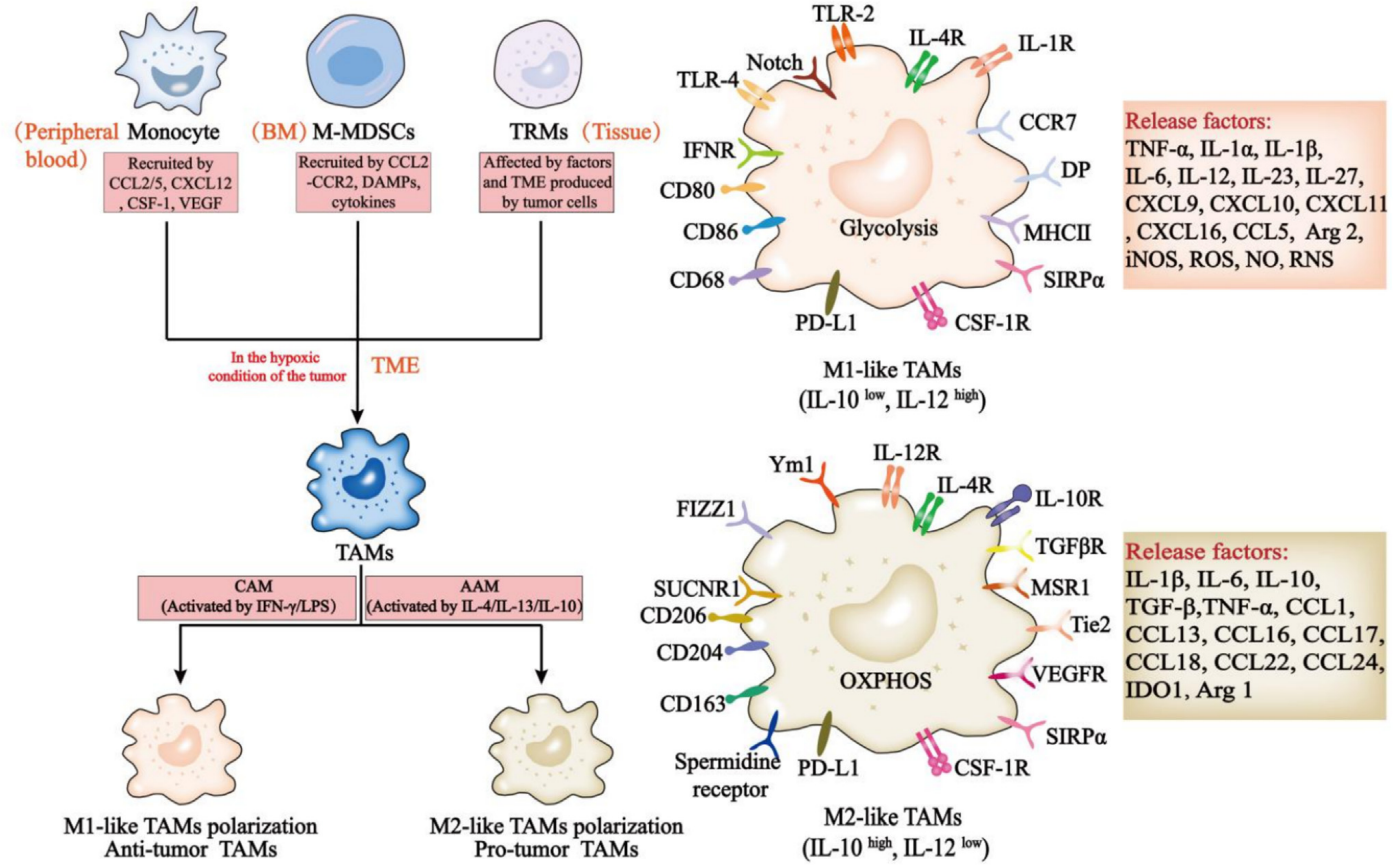
Recruitment and phenotypic characteristics of TAMs in the TME. Monocytes, M-MDSCs and TRMs can infiltrate the TME through different pathways and subsequently differentiate into M1- and M2-like macrophages with different phenotypes. M2-like macrophages constitute a large proportion of the TME. *AAM*, alternative activation of macrophages; *CAM*, classic activation of macrophages; *CCR7*, C–C motif chemokine receptor 7; *CD 68/80/86/163/204/206*, cluster of differentiation; *CCL1/5/9/10/11/13/16/17/18/24*, C–C motif chemokine ligand 1/5/9/10/11/13/16/17/18/24; *CSF-1R*, colony stimulating factor 1 receptor; *DP*, D-prostanoid; *FIZZ1*, found in inflammatory zone 1; *iDOS*, indoleamine-2,3-dioxygenase; *IL-1α/1β/6/10/12/23/27*, interleukin-1α/1β/6/10/12/23/27; *IFNR*, interferon receptor; *iNOS*, inducible nitric oxide synthase; *MHCII*, major histocompatibility complex II; *MSR1*, macrophage scavenger receptor 1 gene; *ROS*, reactive oxygen species; *SIRPα*, signal-regulatory protein α; *SUCNR1,* succinate receptor 1; *TGF-β*, transforming growth factor-β; *Tie2*, TEK receptor tyrosine kinase; *VEGFR*, vascular endothelial growth factor receptor; *Ym1*, chitinase-like protein 3.

TAMs provide a protective niche for cancer growth and invasion at both primary and metastatic sites and are highly heterogeneous [23]. TAMs within the TME originate from bone marrow (BM)-derived monocytes and embryonic-derived tissue-resident macrophages (TRMs) [[24], [25], [26], [27], [28], [29]] (Fig. 2). Hypoxia prompts tumor cells to produce monocyte recruitment factors, such as monocyte chemotactic protein-1 (MCP-1), macrophage colony stimulating factor-1 (CSF-1), and vascular endothelial growth factor (VEGF). Once these monocytes are recruited, the hypoxic microenvironment can effectively retain them by downregulating the expression of the receptors of the factors above [30]. On the other hand, the cytokines and damage associated molecular patterns (DAMPs) in the TME can act on BM progenitor cells to generate monocytic myeloid-derived suppressor cells (M−MDSCs), which are recruited to tumors in a C–C chemokine receptor 2 (CCR2)-dependent manner and subsequently transform into TAMs [[31], [32], [33]], significantly promoting the metastatic growth of breast cancer [32]. In addition, CCR2 can bind to Ly6C^high^ monocytes via the CCR2 receptor, resulting in their recruitment and differentiation into TAMs [34]. Notably, the CCR2 receptor-dependent mode of monocyte recruitment is important for the origin of TAMs. Monocyte-derived TAMs recruited through CCR2 signaling can migrate and differentiate into perivascular macrophages under the regulation of C-X-C motif chemokine ligand 12 (CXCL12) and C-X-C chemokine receptor type 4 (CXCR4), promoting vascular leakage and endoshism [35]. Compared with monocyte-derived macrophages, embryo-derived macrophages may present different phenotypes and functions [[36], [37], [38]]. During cancer progression, resident-tissue macrophage-like TAMs (TRMs) are the first to be impacted by soluble factors produced by tumor cells and other TME damage; under the influence of early inflammatory changes, TRMs assist in the recruitment of BM-derived monocytes and support the production of TAMs [23].

### Phenotypes and clusters of TAMs

Macrophages are highly heterogeneous, and juvenile macrophages (M0) can polarize into two main clusters, M1-like TAMs (M1: proinflammatory stimulation-interferon-γ (IFN-γ)/lipopolysaccharide (LPS)) and M2-like TAMs (M2: anti-inflammatory stimulation-interleukin-4/10/13 (IL-4/IL-10/IL-13)), which have different phenotypes, transcription patterns and functions [[39], [40], [41]]. The activation of TAMs can be classified into classic activation of macrophages (CAM) and alternative activation of macrophages (AAM). M0 macrophages are exposed to cytokines such as IL-12, IFN‒γ, microbe-associated molecular patterns (MAMPs) or other toll‒like receptor (TLR) ligands undergo CAM and enter the M1-like state [42,43]. AAM does not require dual signals and are polarized into an M2-like state inspired by IL-4, transforming growth factor (TGF) -β and prostaglandin E2 (PGE2) [44]. TAMs can be divided into several major clusters according to their phenotype and function. M1-like TAMs can express cluster of differentiation 68 (CD68), CD80, inducible nitric oxide synthetase (iNOS) and secrete IL-1β to exert proinflammatory effects. M2-like TAMs express CD163, CD204, C–C motif chemokine ligand 22 (CCL22) and CCL24, etc. [44,45]. The different functions of M2-like TAMs can be divided into four types: M2a, M2b, M2c and M2d. Although they present different phenotypic markers, gene expression patterns, cytokines and functions, they all exhibit IL-10^high^ and IL-12^low^-like cytokine secretion [26,46,47] (Table 1). Individual M2 subtypes offer new therapeutic targets. For example: M2a is characterized by IL-4/IL-13-induced expression of early angiogenic mediators, offering opportunities to target early-stage vascularization; M2b exhibits a dual cytokine profile and may respond to agents that modulate TLR or immune complex pathways; M2c, enriched in IL-10 and TGF-β, may be amenable to IL-10 neutralization strategies or anti-TGF-β antibodies; and M2d, formed by TLR agonists and adenosine receptor agonists, secretes IL-10 and VEGF, and presents opportunities for antiangiogenic therapy such as anti-VEGF antibodies or tyrosine kinase inhibitors [48,49].

**Table 1:** Phenotypic characteristics and functional summary of M1- and M2-like TAM subsets.

Cell type	Cell subtype	Stimulus factors	Key transcription factors	Phenotypic markers	Release factors	Metabolic profiles	Main function	Ref.
M1	/	LPS, TNF-α, IFN-γ, GM-SCF	NF-κB, STAT1, STAT2, STAT5, IRF3, IRF5, SOCS3, BtK	CD80, CD86, CD68, iNOS, MHC Ⅱ, TLR-2, TLR-4, IL-1R, IL-4R, CCR7, IL-10 ^low^, IL-12 ^high^	ROS, TNF-α, IL-1α, IL-1β, IL-6, IL-12, IL-23, IL-27, CXCL1, CXCL3, CXCL5, CXCL8, CXCL9, CXCL10, CXCL11, CXCL16, CCL15, CCL19, CCL20, CD64, IDO, Arg2, iNOS	Active glycolysis, tryptophan, amino sugar and pyrimidine metabolism, incomplete TCA cycle and succinic acid accumulation, lipid accumulation	①Antitumor effects ②Recruit T cells ③Exert Th1 type reactions ④Pro-inflammation ⑤Mediate ROS-induced tissue damage ⑥Mediate antimicrobial action	[[52], [53], [54], [55], [56], [57]]
M2	M2a	IL-4, IL-13	STAT3, STAT6, P50, IRF4, JMJD3, SMYD3, PPARδ, PPARγ, SOCS2	CD14, CD80, CD86, CD163, CD206, CD209, CD301, MRC1, MHC Ⅱ, Arg1, FIZZ1, Ym1, Ym2, IL-1RΠ, Dectin-1, MMR, IL-10 ^high^, IL-12 ^low^	IL-10, TGF-β, CCL17, CCL18, CCL22, CCL24, Arg1	Active glycolysis, complete TCA cycle, FAO is active	①Mediate anti-inflammatory effects ②Promote tumor cell proliferation and migration ③Regulate tissue repair ④Purge apoptotic cells	[53,54,[58], [59], [60], [61], [62], [63], [64], [65], [66], [67]]
	M2b	immune complex, ligands of TLR, LPS, IL-1β		CD86, MHC Ⅱ, IL-10 ^high^, IL-12 ^low^	TNF-α, IL-1β, IL-6, IL-10, CCL1	Active glycolysis, tryptophan, amino sugar and pyrimidine metabolism, complete TCA cycle	①Participate in immune regulation ②Mediate Th2 type activation ③Promote extracellular matrix formation	[53,54,64,[68], [69], [70], [71]]
	M2c	IL-10, TGF-β, glucocorticoid		CD163, CD206, TLR-1, TLR-8, Arg1, MerTK, Tie2, IL-10 ^high^, IL-12 ^low^	IL-10, TGF-β, CCL16, CCL18, CXCL13	Active glycolysis, accumulate glucose and reduce glutamic acid	①Mediate cellular immune regulation ②Involved in the phagocytosis of apoptotic cells	[53,54,63,64,69,72]
	M2d	ligands of TLR, adenosine receptor ligands, IL-6, LIF		CD14, CD36, CD163, VEGF, TNF-α ^low^, IL-10 ^high^, IL-12 ^low^	IL-10, VEGF	Active glycolysis, remarkable FAO	①Promote tumor development ②Promote angiogenesis and metastasis	[53,54,62,63,73]

However, owing to the significant heterogeneity of macrophages and the expression of overlapping markers, it is difficult to subdivide them through such a strict systematization. Preliminary studies on human tumors have focused mainly on breast cancer, non-small cell lung cancer, liver cancer, glioblastoma, colorectal cancer and renal cell carcinoma. These single-cell RNA sequencing (scRNA-seq) studies revealed the complexity of TAMs and confirmed that the activation of macrophages within tumors may not follow the simple M1/M2 activation model. However, research has shown that in the study of macrophages, the most important aspect is cell function rather than phenotype. A review of recent scRNA-seq data in cancer studies from major journals revealed that seven TAM subsets were preserved in almost all cancer types [50]. On the basis of their signature genes, enriched pathways, and predicated functions, Ma *et al*. [50] proposed naming these TAM subsets interferon-primed TAMs, immune regulatory TAMs, inflammatory cytokine-enriched TAMs, lipid-associated TAMs, proangiogenic TAMs, RTM-like TAMs, and proliferating TAMs. scRNA-seq data revealed that a subpopulation of MER proto-oncogene tyrosine kinase+ (MerTK+) macrophages exist in melanoma. *In vivo* studies revealed that adoptive transfer of MerTK+ macrophages into recipient mice of macrophage-depleted wild-type or Lyz2^cre/+^Ahr^fl/fl^ mice accelerated tumor growth by inhibiting T-cell function. A humanized mouse model of hepatocellular carcinoma (HCC) indicated that HCC-derived oleic acid promoted the transformation of macrophages into M2-like TAMs [51]. These findings challenge the traditional M1/M2 classification and open new directions for precision immunotherapy on the basis of macrophage diversity.

### Endogenous and exogenous factors shape the metabolic profiles of TAMs

**Fig. 3:**
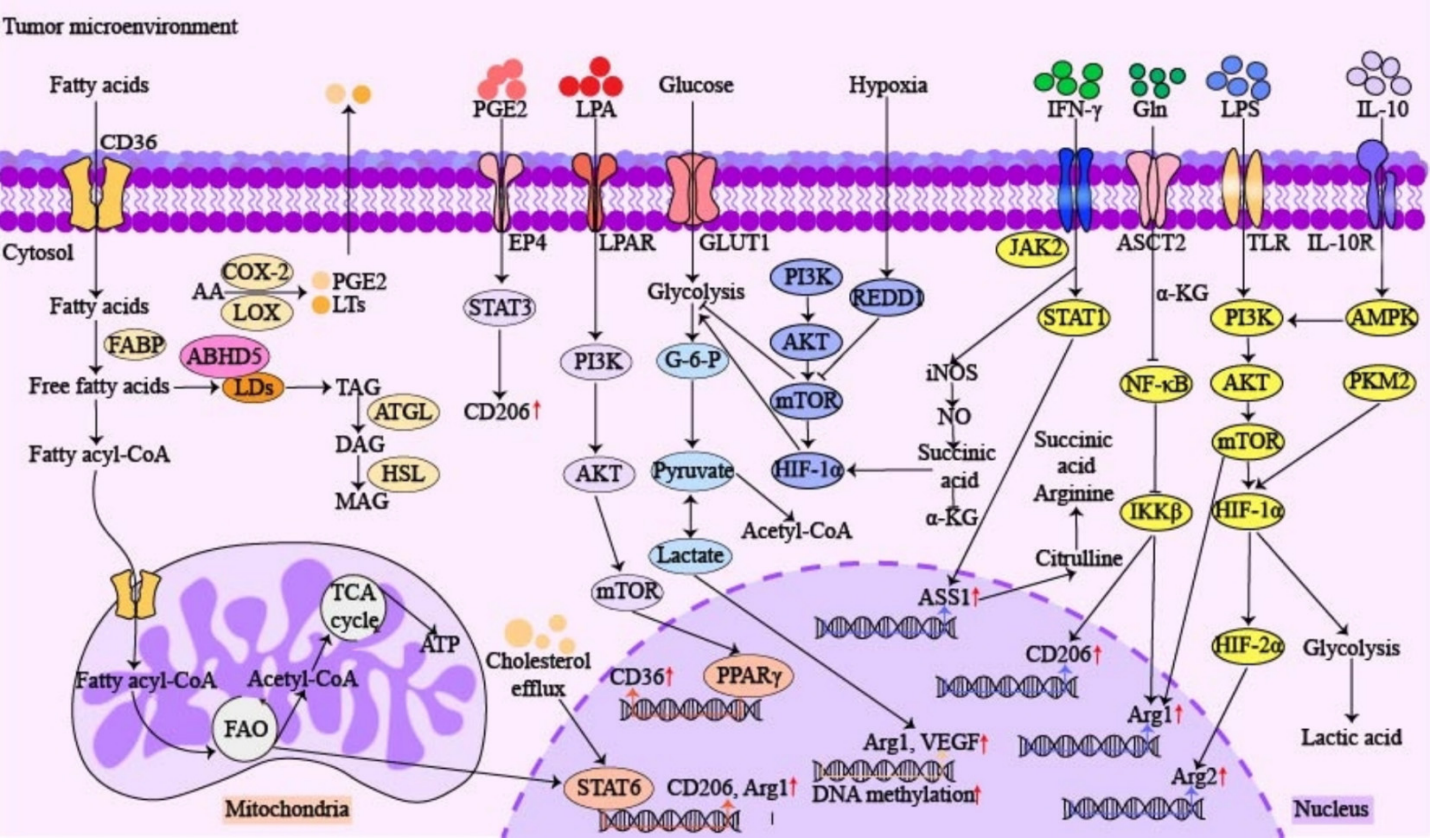
Endogenous and exogenous factors impact the metabolism of TAMs. *AA*, arachidonic acid; *ABHD5*, α/β-hydrolase domain-containing 5; *AKT*, Ak strain transforming; *AMPK*, adenosine 5′-monophosphate (AMP)-activated protein kinase; *ATGL*, adipose triglyceride lipase; *ASS1*, argininosuccinate synthetase 1; *ATP*, adenosine 5′-triphosphate; *CD36*, platelet glycoprotein 4; *CD206*, mannose receptor; *COX-2*, cyclooxygenase-2; *DAG*, diacylglycerol; *G-6-P*, glucose-6-phosphatase G-6-pase; *GLUT1*, facilitative glucose transporter 1; *HIF-1/2α*, hypoxia-inducible factor-1/2α; *HSL*, hormone-sensitive triglyceride lipase; *IKKβ*, iκB kinase β; *FABP*, fatty acid-binding protein; *IL-10/IL10R*, interleukin-10/Interleukin-10 receptor; *JAK2*, janus kinase; *JMJD3*, jumonji domain-containing protein-3; *α-KG*, α-ketoglutarate; *LDs*, lipid droplets; *LPA*, lysophosphatidic acid; *LPAR*, lysophosphatidic acid receptor; *LTs*, leukotrienes; *MAG*, monoacylglycerol; *mTOR*, mammalian target of rapamycin; *NF-κB*, nuclear factor-κB; *PGE2*, prostaglandin E2; *PKM2*, pyruvate kinase M2; *PI3K*, phosphoinositide 3-kinase; *PPARγ*, peroxisome proliferator-activated receptor γ; *PS*, phosphatidylserine; *REDD1*, regulated in development and DNA damage response 1; *STAT1/3/6*, signal transducer and activator of transcription 1/6; *SLC1A5*, solute carrier family 1 member 5; *TCA*, tricarboxylic acid cycle; *TAG*, triacylglycerol; *TLR*, toll-like receptor.

TAMs adopt different metabolic profiles due to the influence of exogenous coordination factors and endogenous signaling pathways [74,75]. In addition to the traditional M1/M2 classification, metabolic profiling analysis of macrophages revealed that although M1 and M2b share metabolic characteristics consistent with inflammatory features, there were key differences in the tricarboxylic acid (TCA) cycle, fatty acid oxidation (FAO), and oxidative phosphorylation (OXPHOS). In addition, metabolic type differences in M2a, M2c and M2d were observed in multiple pathways including hexosamine, polyamine and fatty acid metabolism [73,76,77] (Table 2, Fig. 3).

**Table 2:** Summary of the signaling pathways associated with TAM polarization, metabolism and function.

No.	Signaling pathways	Relevant upstream and downstream components	Polarization direction	Effects on the metabolic processes and functions in TAMs	Ref.
1	PI3K/AKT pathway	TLR4, AKT, Glut1, HK2, receptor tyrosine kinase, HIF-2α, mTORC1	M1	Upregulate glycolysis and lactic acid levels, stimulate VEGF expression	[108,109]
2	SUCNR1/PI3K-AKT /HIF-1α	succinic acid, SUCNR1, PI3K, AKT, HIF-1α	M2	Promote succinic acid levels in the TME	[110]
3	TSC/mTOR pathway	TSC, PI3K, mTORC1, mTORC2, HIF-1α, IRF4, 4E-BP1, Akt, SGK1, PKC-α	M2	Activate regulatory factors of macrophage metabolism, enhance aerobic glycolysis, promote lipid biosynthesis and inhibit autophagy	[[111], [112], [113]]
4	c-Myc pathway	LDHA, PDK1, TGF-β, VEGF	M2	Enhance glycolytic activity, c-Myc upregulate the expression of glutamine transporter	[105,106]
5	Glutamine pathway	Glutamic acid, GPT2, α-KG, UDP-GlcNAc	M2	Promote glutamine metabolism, TCA cycle and UDP-GlcNAc synthesis	[114]
6	IFN-γ/JAK/STAT1	IFN-γ, JAK, STAT1	M1	Promote the M1-like polarization of TAMs	[115]
7	JAK/STAT3	JAK, IL-4, STAT3	M2	Activate oxidative phosphorylation, upregulate PD-L1 expression	[116]
8	JAK/STAT1	IFN-y, JAK, STAT1, NRE1, NAMPT	M1	Activate glycolysis	[115]
9	PIPK3/PPAR	RIPK3, PPAR	M2	Upregulate lipid metabolism	[117]
10	AMPK/HIF-1α	P-AMPK, HIF-1α, Arginine, Ym-1, Fizz1, CD206	M1	Regulate glycolysis and inhibit M2-like TAM polarization	[118]
11	STAT3/Jagged1/Notch	linc00514, STAT3, Jagged1, Notch	M2	Promote the M2-like polarization of TAMs and breast cancer metastasis	[119]
12	Notch pathway	Notch, SOCS3	M1	Promote the M1-like polarization of TAMs	[120,121]
13	Nrf2 pathway	Nrf2, Nqo1/Nqo2, IL-6, IL-1β	M2	Inhibit the expression of inflammatory cytokine genes in M1-like TAMs, itaconate plays an immunosuppressive role through the NRF2-dependent pathway	[122]
14	TLR/NF-κB	PAMP, TLR, α-KG, PHD, NF-κB, IκBα, HIF-1α	M2	α-KG promotes immunosuppression by interfering with the NF-κB pathway and prevents M1-like polarization of TAMs, participate in glucose metabolism	[109,[123], [124], [125]]
15	STAT1/ASS1	IFN-γ, LPS, JAK2, STAT1, ASS1	M1	Promote citrulline depletion, mediate the activation of inflammatory signals, control inflammatory macrophages activation	[126]
16	IL-4/STAT6	IL-4, STAT6, PPARG, PPARGC1B	M2	Increase mitochondrial biogenesis and epigenetic reprogramming toward fatty acid oxidation	[127]

Glucose metabolism: Glucose is considered the main energy source in TAMs, and TAMs compete with tumor cells for glucose and undergo highly active glycolysis13 [[78], [79], [80]]. Like in tumor cells, in M1-like TAMs, glucose metabolism and glycolysis are increased, the pentose phosphate pathway (PPP) is increased, OXPHOS is decreased, and glucose uptake is strongly increased [81]. M2-like TAMs exhibit a complete TCA cycle, enhanced OXPHOS, and reduced glycolysis and PPP [[82], [83], [84]]. In M2-like TAMs, the PI3K/mTOR axis promotes glucose consumption and metabolism [[85], [86], [87]], c-Maf promotes the TCA cycle and UDP-GlcNAc biosynthesis [88], and hypoxia-inducible factor-1α (HIF-1α) promotes VEGF expression and lactate-induced M2-like polarization [89]. Moreover, compared with M2a, M2c and M2d, M2b has a broader range of metabolic adaptations. M2b, M2c and M2d macrophages exhibit increased glycolysis compared with M2a macrophages according to changes in metabolite levels, and M2a macrophages tend to increase the TCA cycle and OXPHOS [90]. During the polarization process, the PPP of M2a and M2b increases, but that of M2c decreases [73].

Lipid metabolism: TAMs from human and mouse tumor tissues are rich in lipids [91], and both M1- and M2-like TAMs increase fatty acid synthesis, which is impacted by metabolic pathways in TAMs and related factors in the TME. Studies have shown that the peroxisome proliferator activated receptor (PPAR) signaling pathway promotes FAO in TAMs [92,93]. Increased FAO promotes the expression of mannose receptor C-type 1 (MRC1), transglutaminase 2 (TGM2), arginase 1 (Arg1) and other genes through signal transducer and activator of transcription 6 (STAT6) phosphorylation to maintain M2-like TAMs [91]. Despite the increased FAO level, some TAMs accumulate lipids in the cell to maintain their own metabolic and immune effects. In these TAMs, the expression of various proteins involved in lipid metabolism, such as adipose triglyceride lipase (ATGL), self-hydrolase domain 5 protein (ABHD5), and fatty acid binding protein (FABP), is abnormally regulated [94,95]. Research on M2-like TAMs has fshown that fatty acid synthesis (FAS) in M2c macrophages continuously increases and is closely related mainly to tissue repair function [96]. In addition, compared with other phenotypes, M2b is more inclined toward the general pattern of lipid consumption [73].

Amino acid metabolism: Tumor cells compete with TAMs in the microenvironment for amino acids. Enzymes involved in amino acid metabolism play important roles in the metabolic mechanism of TAMs. Arg1 and inducible nitric oxide synthase (iNOS) determine the utilization of arginine pathways and the polar direction in TAMs [97]. M1-like TAMs upregulate and highly express iNOS, which can metabolize arginine to NO and citrulline [98]. Unlike M1-like TAMs, M2-like TAMs use the upregulated expression of Arg1, and pharmacological inhibition of Arg1 does not synergize with anti-programmed death 1 (PD-1) therapy [99], inducing tumor-supporting factors such as polyamines and ornithine through the polyamine metabolic pathway [100,101]. In addition to arginine, M2-like TAMs tend to use glutamine and fatty acids as energy sources, and the catabolism of glutamine plays a key role in supporting M2-like polarization [97,98,102]. Although only M1-like TAMs accumulate alanine and asparagine, aspartic acid accumulates in all polarization phenotypes except M2d [73]. Myelocytomatosis viral oncogene homolog (c-Myc) plays an important role in amino acid metabolism by upregulating the expression of glutamine transporters, which participate in M2-like TAM polarization [[103], [104], [105], [106]]. The increase in the intracellular levels of succinate in M2b, M2c and M2d is caused by HIF-1α, and the accumulation of HIF-1α also drives the synthesis of nitric oxide (NO) by activating the aspartate-arginine-succinate shunt, which intersects with the TCA cycle and uses oxaloacetic acid to produce aspartic acid to promote the synthesis of NO, further promoting the utilization of aspartic acid and citrulline [90,107].

## Diverse roles of TAMs in the TME

### Promotion of tumor cell vascularization

**Fig. 4:**
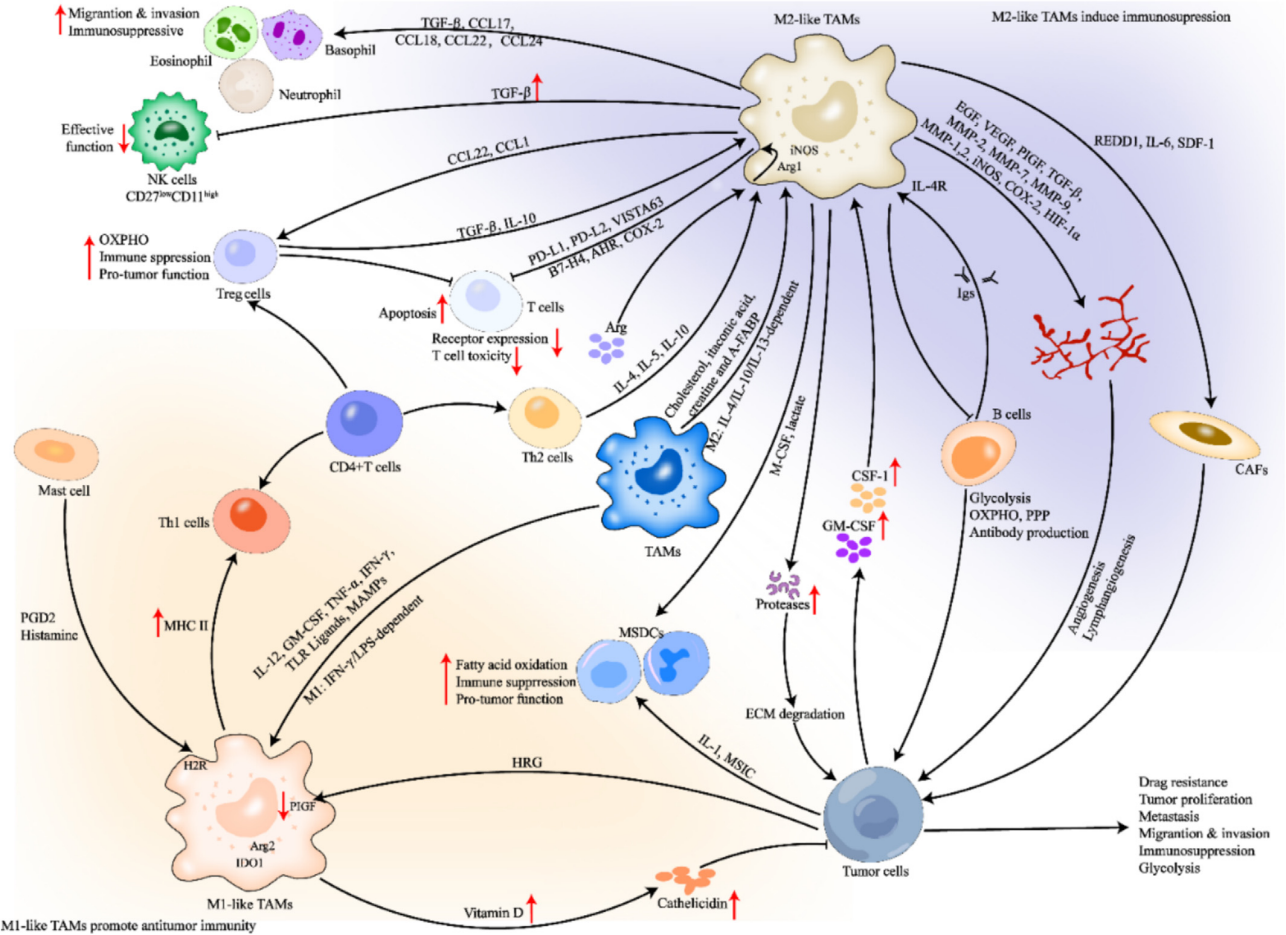
Diagram of TAMs interacting with other immune cells in TME. *A-FABP*, adipocyte fatty acid-binding protein; *AhR*, aryl hydrocarbon receptor; *CCL17/18/22/24*, chemokine ligand 17/18/22/24; *COX-2*, cyclooxygenase-2; *GM-CSF*, granulocyte–macrophage colony-stimulating factor; *IL-4/10/12/13*, interleukin-4/10/12/13; *IL-6*, interleukin-6; *LPS*, lipopolysaccharides; *MAMPs*, metabolism-associated molecular patterns; *MMP-1/2/7/9*, matrix metalloproteinase-1/2/7/9; *SDF-1*, stromal cell-derived factor-1; *PDGF*, platelet-derived growth factor; *TNF-α*, tumor necrosis factor-*α*; *Th1 cell*, T helper 1 cell.

The main mechanism of nutritional support of TAMs to malignant cells is neoangiogenesis, which depends on the recruitment or activation of endothelial cells by TAM derivatives M2-like TAMs are enriched in hypoxic regions with insufficient blood supply and promote tumor progression mainly by producing factors such as VEGF, platelet-derived growth factor (PDGF), TGF-β and fibroblast growth factor (FGF) to support tumor angiogenesis and inhibit antitumor immunity [[128], [129], [130], [131]]. In addition, M2-like macrophages can express a series of enzymes, including matrix metalloproteinase-2/7/9/12 (MMP-2/7/9/12) and cyclooxygenase-2, which are involved in the regulation of angiogenesis [132,133]. HIF-1α is also a key driver of the angiogenic phenotype of TAMs, and tumor-derived lactic acid stabilizes HIF-1α expression and further induces VEGF production in TAMs [[134], [135]]. In addition, a second break in the TCA cycle after succinic acid formation leads to increased expression of HIF-1α [136]. MMP188 and cathepsin 189 expressed by TAMs can support tumor angiogenesis through extracellular matrix degradation [135] (Fig. 4). Blocking VEGF expression in TAMs not only inhibits glycolysis but also adversely affects neovascularization, thereby reducing the infiltration of TAMs in the TME [137,138].

### Promotion of tumor cell invasion

TAMs can stimulate tumor cell proliferation, migration and genetic instability. Pathological staining of tumor samples revealed that epithelial‒mesenchymal transition (EMT) active sites, such as tumor margins, are often accompanied by the infiltration of many TAMs [139]. M2-like TAMs express high levels of fibronectin, which plays an important role in tissue repair and cell motility [[140], [141], [142]]. Moreover, secreted protein acidic and cysteine rich (SPARC), an acidic cysteine-rich secreted protein synthesized by TAMs, is conducive to the interaction of fibritin and velentin with tumor cells through integrins, generating traction along extracellular matrix (ECM) fibers and promoting tumor metastasis [143]. TAMs secrete a variety of proteolytic enzymes, including cathepsins, MMPs, and serine proteinases, which mediate ECM degradation and are important components of cell–ECM interactions [144,145] (Fig. 4). Cytokines produced by tumor cells also promote the differentiation process of TAMs, thus forming a positive feedback loop between TAMs and EMTs [146]. Colonizing factor 1 (CSF1)-based interactions between cancer-associated fibroblasts (CAFs) and bone marrow-derived macrophages inhibit tumor progression through drug ablation and subpopulation-specific CSF1/ colony stimulating factor 1 receptor (CSR1R) [147].

### Interaction with other immune cells in the TME

Crosstalk between TAMs and other immune cells is an important aspect of how TAMs affect tumor immunity. The immunosuppression mediated by TAMs is related mainly to the types and functions of T cells infiltrating the TME. M2-like TAMs can inhibit cytotoxic T cells (CTLs) through at least three different mechanisms: (1) direct inhibition of cell‒cell contact via checkpoint suppressor molecules; (2)production of inhibitory cytokines; and (3) regulation of the metabolic environment by the consumption of arginine and production of reactive oxygen species (ROS) [148,149]. In addition, TAMs can affect the immune function of CTLs in indirect ways, such as by recruiting immunosuppressive cells, restricting the antigen-presenting function of dendritic cells (DCs), and regulating the vascular structure and extracellular matrix to block the recruitment of CTLs to the microenvironment [149] (Fig. 4). TAMs express high levels of immune checkpoint ligands, such as programmed death ligand-1 (PD-L1), PD-L262, and V-type immunoglobulin domain-containing suppressor of T cell activation (VISTA), which directly inhibit T-cell activity [[148], [150]]. Moreover, M1-like TAMs highly express major histocompatibility complex II (MHC II) and can regulate and promote T helper 1 (Th1) cellimmune responses by presenting antigens to T cells [149]. Targeting the reprogramming of monocytes and macrophages in breast cancer promotes antitumor immune responses mediated by CD8+ T cells [151].

TAMs support tumor progression primarily by (1) indirectly increasing the availability of selected nutrients in the TME, (2) providing nutritional signals to malignant cells, and (3) mediating powerful immunosuppressive functions [152].TAMs derived from resected human glioblastoma, as well as TAMs derived from the mouse TME and immature bone marrow cell populations, consume nonessential amino acids necessary for the efficient function of T cells, such as, glutamic acid, glutamine, serine, and cysteine, strongly affecting the immune surveillance role of the TME [[153], [154], [155], [156], [157]]. TAMs directly inhibit CD8+ T-cell proliferation by metabolizing L-arginine via Arg1, iNOS, and oxygen radicals [158], promoting tumor progression [159,160], and leading to intense dysfunction of T and natural killer (NK) cells [161,162]. Studies have shown that glutamine-deprived TAMs secrete IL-23 to reduce the survival and immunosuppressive function of regulatory T cells (Tregs) [163]. These observations further confirm that TAMs and tumor cells interact both metabolically and immunologically (Fig. 4).

## Amino acid metabolic reprogramming in TAMs induces an immunosuppressive TME

### Tryptophan metabolic reprogramming promotes the M2-like polarization of TAMs

Both TAMs and tumor cells can activate indoleamine 2,3-dioxygenase (IDO) or tryptophan 2,3-dioxygenase (TDO2) and inhibit T-cell function through tryptophan consumption and the accumulation of the tryptophan metabolite kynurenine (Kyn) to reshape the immunosuppressive environment [164,165] (Table 3). Kyn accumulation promotes tumor proliferation and metastasis while promoting the M2-like polarization of TAMs through the aryl hydrocarbon receptor (AhR).

**Table 3:** Amino acid metabolism in TAMs and its impacts.

No.	Amino acids	Source	Transporter	Key enzyme	Key factors and signaling pathways in TAMs	Effects on the metabolism and functions in TAMs	Impacts on tumors	Other impacts	Ref.
1	Tryptophan	TME	LAT1	IDO1	Kyn metabolism NF-κB signaling pathway	Increase IDO1 expression, Kyn produced by tryptophan metabolism binds to AhR in TAMs and promotes M2-like TAM polarization	IDO1/TDO2 overexpression consume Trp in the TME, resulting in Kyn acting on AhR in TAMs	Inhibit CD8+ T-cell function and stimulate Treg cells	[164–174,263]
2	Arginine	TME	MCAT2	Arg1 iNOS	PI3K/AKT/mTOR/HIF-1α	Increase Arg1 expression, promote M2-like TAM polarization, produce ornithine and polyamines, and supports tumors	Promote the proliferation and angiogenesis of tumors	Energy production and differentiation are disrupted and TCR is impaired in CD8+ T cells	[[198], [199], [200], [201], [202], [203], [204], [205], [206], [207], [208], [209], [210]]
3	Glutamine	TME and endogenous synthesis	ASCT2 LAT1	GS GSL GDH SDH	SUCNR1/PI3K/HIF-1α NF-κB signaling pathway STAT3/AMPK/REDD1/mTORC1	Promote glutamine metabolism, the TCA cycle and UDP-GlcNAc synthesis; a high α-KG/succinic acid ratio promotes M2-like TAM polarization	Enter the TCA cycle, the high expression of GS in TAMs promote tumor angiogenesis and invasiveness	Inhibit CD8+ T-cell function	[80,177,178,181,182,184,185,187,264]
4	Serine	TME and endogenous synthesis	ASCTs	PHGDH	One-carbon metabolism JAK1/STAT1 mTOR signaling pathway	Serine metabolism regulates the polarization of M2-like TAMs and JAK-STAT1 signaling through IGF1-dependent p38 activation	PHGDH inside tumors can influence the polarization of macrophages to form M2-like TAMs by secreting IGF1	Inhibit CD8+ T- cell function	[219,223,224]
5	Branched- chain amino acids	TME	LAT1 MCT1	BCAT1 BCAT2	NF-κB signaling pathway	BCKAs, the degradation product of BCAA metabolism, regulate the phagocytosis of M2-like TAMs through the NF-κB pathway	Enter the TCA cycle, high levels of succinic acid activate NRF2 signaling, enhancing the antioxidant level	Inhibit glycolysis in CD8+ T cells and secrete factors that promoting M2-like polarization of TAMs	[227–229,231,[233], [234], [235], [236],^265^]
6	Methionine	TME and endogenous synthesis	LAT4	MAT2A	One-carbon metabolism Methionine cycle NF-κB signaling pathway	Enhance the secretion of TNF-α by TAMs, decrease the Arg1 level in TAMs, and promote M1-like polarization of TAMs	Induce the activation of the NF-κB signaling pathway, increase the expression of tumor LAT4, and promote proliferation	Tumors compete with CD8+ T cells for methionine; inhibits CD8+ T-cell function	[238,[243], [244], [245], [246], [247]]
7	Taurine	TME	TauT1	LCMT1 PME1	One-carbon metabolism NF-κB signaling pathway	Promote the expression of M2-like TAMs markers such as CD206, Arg1 and IL-10 levels	Taurine secreted by TAMs helps tumor cells resist iron death	Inhibits CD8+ T-cell function	[[248], [249], [250], [251], [252], [253],[255], [256], [257]]

**Fig. 5:**
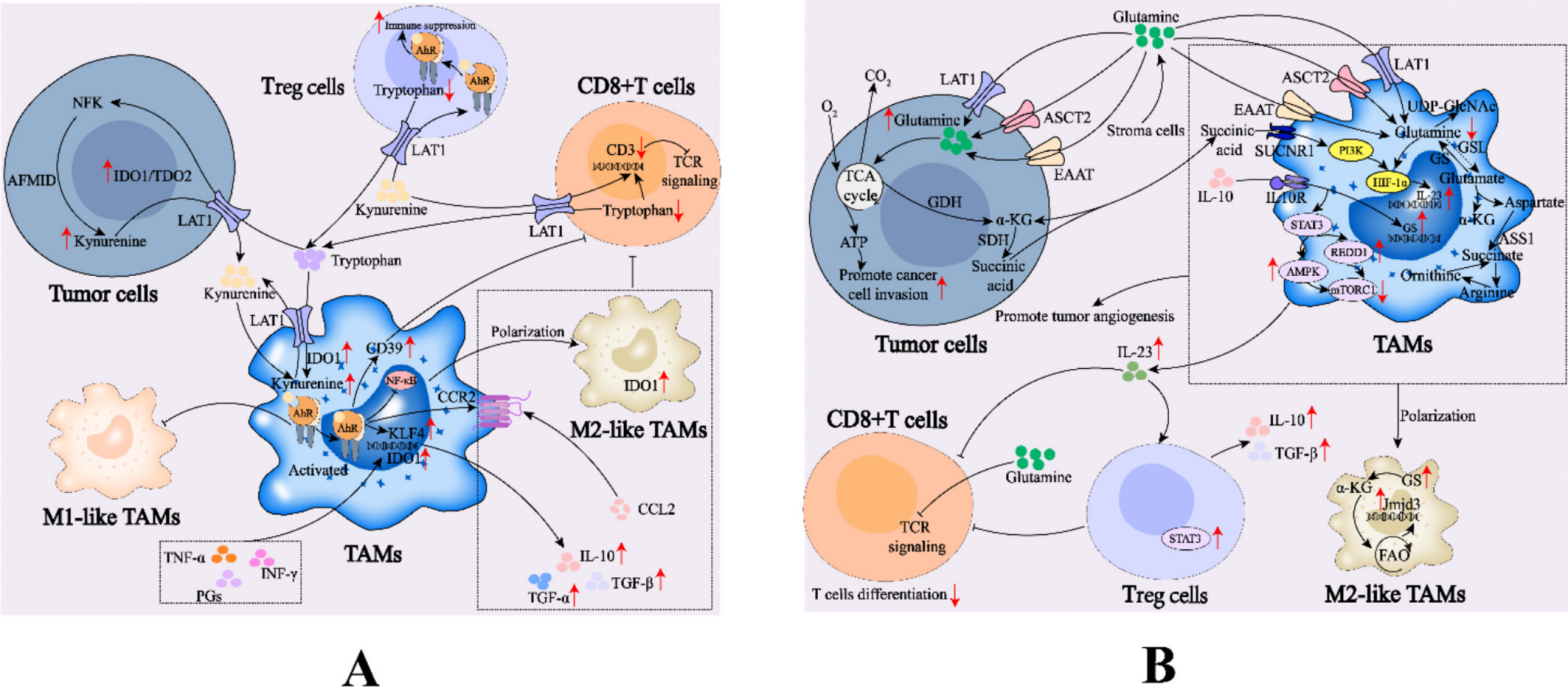
A. Role of tryptophan metabolic reprogramming in TAMs. B. Role of glutamine metabolic reprogramming in TAMs. *AhR*, aryl hydrocarbon receptor; *ASCT2 (SLC1A5)*, solute carrier family 1 member 5; *CCR2*, C–C motif chemokine receptor 2; *CD3*, gamma subunit of the T-cell receptor complex; *EAAT*, excitatory amino acid transporter; *GDH*, glutamate dehydrogenase; *GLS*, glutaminase; *GS*, glutamine synthase; *IL-23*, interleukin-23; *IL-10*, interleukin-10; *IDO1*, indoleamine 2,3-dioxygenase 1; *Jmid3*, jumonji domain-containing protein-3; *KLF4*, krüppel-like factor 4; *LAT1*, L-type amino acid transporter 1; *REDD1*, regulated in development and DNA damage-response 1; *SDH*, succinate dehydrogenase; *STAT3*, signal transducer and activator of transcription 3; *TCR*, T-cell antigen receptor; *TGF-α*, transforming growth factor-α; *mTORC1*, mechanistic target of rapamycin complex 1; *UDP-GlcNAc*, uridine diphosphate n-acetylglucosamine.

TAMs can directly consume tryptophan through the upregulation of IDO1 expression, thus producing immunosuppressive effects in the TME. The tryptophan metabolite Kyn accumulates in the TME and promotes the M2-like polarization of TAMs through AhR signaling in TAMs (AhR is a sensor of tryptophan metabolites and an effective regulator of immunity), and AhR is enriched in human TAMs [166]. M2-like TAMs expressing IDO inhibit the immune response by promoting Treg function (Fig. 5, A). When activated, the AhR of TAMs can drive them to acquire an immunosuppressive phenotype, induce the expression of the immunosuppressive cytokine IL-10, and drive the expression of TGF-α, TGF-β and Arg1 [167], thereby inhibiting immune function and promoting tumor immune escape [168]. In addition, the metabolite Kyn can effectively inhibit the T-cell immune response through AhR, transform naive T cells into Treg cells, and inhibit Th17 cell differentiation [169,170]. A phase II trial revealed excessive infiltration of IDO1-expressing TAMs and a significant increase in the Kyn/tryptophan ratio in most patients who did not respond well to the combination of rhythmic cyclophosphamide and pembrolizumab [171].

Kyn produced by tumor cells that metabolize tryptophan can affect the immunophenotype of TAMs and further affect T-cell function (Fig. 5, A). Takenaka *et al.* demonstrated that glioblastoma cells expressing IDO1-produced Kyn can activate the AhR of TAMs, which induces the expression of Kruppel-like factor 4 (KLF4) after AhR activation, inhibits nuclear factor-κB (NF-κB) activation and promotes polarization toward M2-like TAMs [172]. The activation of AhR in TAMs promotes the expression of CCR2 and drives the recruitment of TAMs in response to CCL2; it is difficult for AhR-deficient TAMs to be recruited to the TME [172]. AhR can drive the expression of the nucleotidase CD39 in TAMs to regulate T-cell immunity, and TAMs promote CD8+ T-cell dysfunction by cooperating with CD73 to produce adenosine [172]. In esophageal squamous cell carcinoma, the overexpression of TDO2 is more obvious than that of IDO1, and the overexpression of TDO2 promotes the degradation of tryptophan, leading to increased Kyn secretion into the TME. Kyn can not only activate AhR on tumor cells to promote their proliferation and invasion but also induce M2-like polarization of TAMs by activating the AKT/GSK3β signaling pathway [173]. In pancreatic ductal adenocarcinoma (PDAC), the tryptophan metabolism-AhR pathway significantly affects M1/M2-like polarization and immune escape induction in tumors by inhibiting immune function and promoting immune escape [166], and inhibiting AhR can increase T-cell function and inhibit tumor growth [166]. Tryptophan can be degraded to activate AhR in TAMs, thereby promoting PDAC tumor progression by inhibiting the function of CD8+ T cells [166]. In IDH-mutant gliomas, the immunosuppressive function of TAMs driven by tryptophan catabolism along the Kyn pathway can be reversed by the drug-mediated suppression of tryptophan metabolism and AhR [174]. scRNA-seq data revealed that a subpopulation of MerTK+ macrophages exist in melanoma. The phosphorylation and activation of MerTK depend on AhR-mediated ALK and LTK ligand 1 (ALKAL1) transcription, thereby enhancing the phagocytosis of MerTK+ macrophages and polarizing them into an immunosuppressive phenotype. *In vitro*, the expression of Arg1, CD206 and PD-L1 increased. *In vivo* studies revealed that the adoptive transfer of MerTK+ macrophages into recipient mice of macrophage-depleted wild-type or Lyz2^cre/+^Ahr^fl/fl^ mice accelerated tumor growth by inhibiting T-cell function [175].

AhR-deficient TAMs exhibit a proinflammatory phenotype, leading to an increase in IFN-γ*+* and CD8+ T cells in tumors, enhancing the effect of immunotherapy. In addition, human monocyte-derived macrophages expressing IDO1 have been shown to consume tryptophan to inhibit T-cell proliferation and viability *in vitro*, and IDO1 macrophage expression has also been shown to promote immune tolerance to apoptotic cells [164], with IDO in TAM inhibitor pretreatment maintaining T-cell proliferation [169,170]. The increased metabolic demand for tryptophan in tumors and the accumulation of the metabolite kynurenine in the TME are among the reasons for the M2-like polarization of TAMs. For different phenotypes, the M2b phenotype tends to consume tryptophan, leading to the accumulation of Kyn [73]. These findings highlight the critical role of AhR in TAM polarization, function, and tumor immune evasion in tryptophan metabolic reprogramming, where the inhibition of AhR activity can trigger immune responses, making AhR a potential target for tumor immunotherapy.

### Glutamine metabolic reprogramming results in M2-like polarization of TAMs

Glutamine and glutamate provide energy for TAMs within the TME, and glutamine released into the TME provides power for tumor cells [176] (Table 3). An increase in glutamine anabolism and catabolism is conducive to inducing the M2-like polarization of TAMs [79,177], and glutamine and succinic acid can act as opposite mediators of M2-like TAMs [178].

Glutamine synthesis mediated by glutamine synthase (GS) expressed by TAMs is one of the basic steps in the differentiation of M2-like TAMs (Fig. 5, B). Additionally, M2a and M2c can be distinguished by glutamine consumption, and M2a relies heavily on glutamine for the production of N-glycans and arginine flux through ornithine for collagen production [179,180]. In the context of glutamine deficiency, macrophages tend to overexpress GS at the cellular level to supplement glutamine [181]. In addition, glutamine synthesis is upregulated, and glutamine is secreted into the TME in heterogeneous stromal cell populations, such as cancer-associated fibroblasts, in tumors [182]. In triple-negative breast cancer (TNBC), both solute carrier family 1 member 5 (ASCT2) and large neutral AA transporter 1 (LAT1) are overexpressed. High expression of ASCT2 can increase glutamine uptake and metabolism, thereby activating the mechanistic target of rapamycin complex 1 (mTORC1) nutritional sensing pathway [183]. Compared with other subtypes of breast cancer, ASCT2 is more sensitive to treatments that target glutamine decomposition. Clear cell renal cell carcinoma competes with TAMs for glutamine and induces the secretion of IL-23 by TAMs through the activation of HIF-1α, thereby promoting the proliferation of Treg cells and the production of IL-10/TGF-β. Upregulated GS expression in TAMs in Lewis lung cancer (LLC) mouse models and glioblastoma patients induces tumor-promoting M2-like polarization of TAMs [178], and specific GS knockdown reverses LLC-associated TAM polarization into an antitumor phenotype and attenuates metastasis [178]. Oh *et al*. [182] found that blocking glutamine metabolism can enhance tumor-specific immunity by inhibiting the transcription of CSF3 in tumor cells, which decreases the generation and attraction of MDSCs and stimulates the creation of proinflammatory TAMs in TNBC. After GS deletion in TAMs, the percentage of cytotoxic CD8+ T cells has been shown to increase by 75 % [178]. The inhibition of glutamine metabolism, which specifically targets the key metabolic enzyme glutaminase (GLS) 1, has been shown to reshape the TME by inducing the M1-like polarization of TAMs and increasing cytotoxic T lymphocyte (CTL) infiltration in cholangiomas [178,184]. In the TME, the inhibition of GS by methionine sulfoxide (a GS inhibitor) leads to a strong increase in glutamate within TAMs, glutamine metabolism is rerouted to succinate synthesis via gamma-aminobutyrate (GABA), and M2-like TAMs are repolarized to M1-like TAMs. Targeting glutamate-aminoligase (GLUL)within TAMs re-educates M2-like TAMs to M1-like TAMs, stimulating T effectors to accumulate succinate and HIF-1α [178].

Glutamine catabolism also plays a crucial role in TAM phenotypic transformation (Fig. 5, B), and glutamine metabolism in M2-like TAMs is essential for supporting TCA cycle activity and providing substrates for N-glycosylation, thereby enabling the glycosylation of M2-like TAM-related proteins [114]. The results revealed that glutamine deprivation can reduce the expression of markers of M2-like TAMs and weaken the TCA cycle; however, the M1-like polarization of TAMs is not affected [178]. It has been reported that α-ketoglutaric acid (α-KG) produced by glutamine catabolism enters the TCA cycle and facilitates the substitution activation of M2-like TAMs, whereas succinic acid promotes the reeduction of M2-like TAMs into M1-like TAMs [178]. α-KG enhances the M2-like polarization of TAMs through JMJD3-dependent epigenetic reprogramming [185], and glutamine metabolism can regulate IKβ activity and limit the M1-like polarization of TAMs by blocking the NF-κB pathway through an α-KG-PHD-dependent mechanism [186]. A high α-KG/succinic acid ratio promotes the M2-like polarization of TAMs, whereas a low ratio enhances classically activated M1-like TAMs [75,79]. The production of glutamine-derived α-KG also leads to the polarization of macrophages into M2-like TAMs by regulating the epigenetic reprogramming of genes in M2-like TAMs [79,187]. M2-like TAMs promote α-KG accumulation by inhibiting α-KG dehydrogenase activity, increasing the α-KG/succinic acid ratio, and further inhibiting the expression of inflammatory genes by inhibiting NF-κB [79,188]. α-KG supplementation can inhibit M1-like activation in a rat alveolar macrophage line [189]. In addition to entering the TCA cycle, glutamine also contributes to nucleotide and uridine diphosphate n-acetylglucosamine (UDP-GlcNAc) synthesis to support protein folding and transport [190]. In M2-like TAMs, the glutamine pathway to UDP-GlcNAc is particularly enhanced because this molecule represents the building block of synthesis of the glycosylated portion of the lectin/mannose receptor, which, in its highly glycosylated form, is one of the polarization markers of M2-like TAMs [191]. Its downstream product α-KG is the metabolic center of the TAM immune response, and glutamine can enter the TCA cycle and hexosamine pathway through the production of α-KG and promote the M2-like polarization of TAMs [178,192]. When OXPHOS is inhibited in tumor cells, glutamine consumption increases to drive the TCA cycle, and glutamine is converted to α-KG by glutamate dehydrogenase or amino transferase [193]. Succinic acid, a downstream product of α-KG, is an intermediate in the TCA cycle in mitochondria, and its levels are primarily controlled by succinate dehydrogenase (SDH), which is considered a tumor suppressor [194]. Genetic or somatic mutations, as well as the inhibition of SDH, lead to tumor formation and the accumulation of succinic acid in tumor cells [195]. The uptake of glutamine by tumor cells induces a glutamine-based axis, and the gamma-aminobutyric acid shunt pathway also increases succinic acid levels [196]. Tumor-derived succinic acid is taken up via the succinic acid receptor 1 (SUCNR1) and leads to the polarization of TAMs into tumor-promoting forms via the SUCNR1/PI3K/HIF-1α signaling pathway to enhance tumor metastasis, which is also mediated by autocrine succinic acid [110].

### Arginine metabolic reprogramming promotes the M2-like polarization of TAMs

**Fig. 6:**
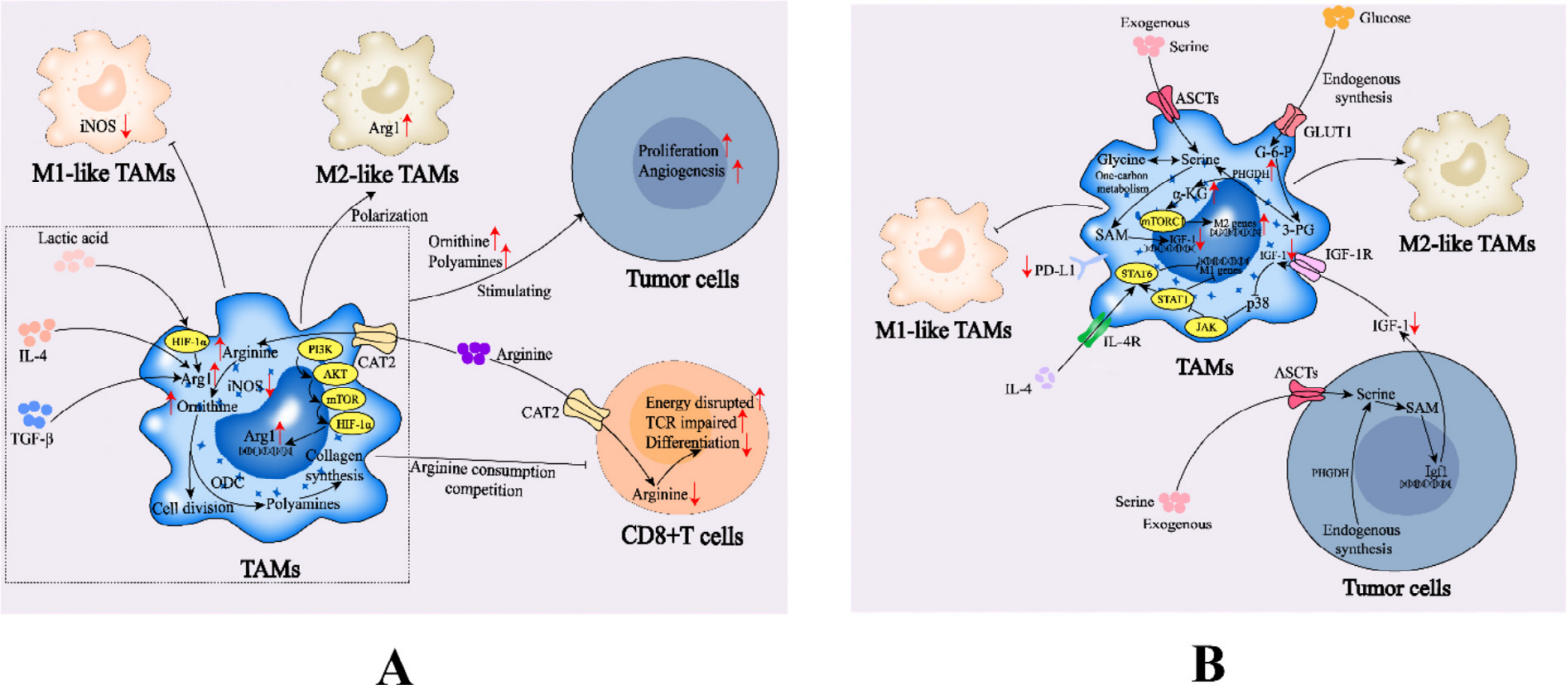
A. Role of arginine metabolic reprogramming in TAMs. B. Role of serine metabolic reprogramming in TAMs. *CAT2 (SLC7A2)*, solute carrier family 7 member 2; *IL-4*, interleukin-4; *IGF-1*, insulin-like growth factor 1 receptor; *JAK*, Janus kinase; *ODC*, ornithine decarboxylase; *3-PG*, 3-phosphoglyceric acid; *SAM*, S-adenosylmethionine.

Changes in arginine metabolism caused by tumor progression induce M2-like polarization of TAMs (Fig. 6A, Table 3). A previous study indicated that creatine can be synthesized from arginine via the transfer of guanidinoacetic acid, which is consumed in large amounts in both M1 and M2b, and ornithine accumulates in M1, M2a, M2c and M2d, whereas urea accumulates in M1, M2a, M2b and M2c73 [90]. The dependence of tumor cells on arginine is different from that of normal cells according to the condition of arginine succinic synthase 1 (ASS1). The seizure of arginine by tumor cells in the TME results in a deficiency of arginine in the microenvironment, thus inhibiting the activity of antitumor cells and leading to immunosuppression. In the early stage of tumor formation, most TAMs are M1. With the progression of tumors, tumor cells secrete anti-inflammatory cytokines such as IL-4 and TGF-β to regulate the polarization of TAMs toward the M2-like phenotype and simultaneously regulate the metabolism of arginine in macrophages; that is, they upregulate Arg1 expression and suppress the activity of iNOS, resulting in excessive production of ornithine and reduced production of NO and citrulline [197,198]. TAMs preferentially metabolize arginine through the Arg1 pathway, forming a competitive relationship with the iNOS pathway, resulting in NO deletion to further prevent M1-like repolarization to M2-like TAMs [[199], [200], [201], [202]]. Studies have shown that TAMs can regulate HIF-1α through the PI3K/AKT/mTOR pathway, upregulate Arg1 expression, and enhance arginine metabolism [87,203]. Lactic acid in the TME, a byproduct of aerobic or anaerobic glycolysis, increases the expression of Arg1 in TAMs by stabilizing HIF-1α [134].

In addition to affecting their phenotype and functions, TAMs can induce the formation of the TME by influencing immune cells. Arg1 activity in TAMs can induce protumor phenotypes and reduce T-cell proliferation and cytokine production, leading to immunosuppression in the TME [204]. High arginine levels are essential for the survival and proliferation of T cells [86,205]. M2-like TAMs can consume arginine in the TME by increasing Arg1 expression, inhibiting T-cell NO and protein synthesis, affecting T-cell receptor (TCR) function [206] and T-cell differentiation, and interfering with antitumor activity [207,208]. In addition to their role in immune cells, TAMs that increase Arg1 consumption of arginine can stimulate arginine metabolism in nearby tumor cells [205] and TAMs. With the progression of tumors, tumor cells regulate the metabolism of arginine in TAMs by secreting anti-inflammatory factors such as IL-4 and TGF-β; that is, they upregulate Arg1 and downregulate the activity of iNOS, resulting in the excessive production of ornithine and decreased production of NO [209]. A decrease in NO reduces the killing effect of NO on tumor cells. In addition, the downstream product of arginine metabolism in macrophages, creatine, inhibits the induction of immune-related molecules such as iNOS by inhibiting IFN-γ-JAK-STAT1 transcription factor signaling while enhancing M2-like activation by promoting chromatin remodeling in support of Arg1 expression for IL-4-STAT6 activation [210]. In breast cancer, collagen-producing TAMs restrict the antitumor CD8+ T cell response via a dual mechanism: they generate a physically stiff matrix that is inaccessible to CD8+ T cell and they deplete l-arginine, which is required to support T cell proliferation [211]. Genetic deletion of Arg1 in TAMs in an autochthonous mouse model of pancreatic cancer increased CD8+ T cell infiltration and delayed invasive disease [212]. Genetic deletion of Arg2 in cancer cells in an orthotopic transplant model of obesity-driven PDAC suppressed tumorigenesis [213]. TAM-derived ornithine is beneficial for the proliferation of tumor cells and can be converted by ornithine decarboxylase (ODC) into polyamines, including humutine, spermidine and spermidine, thereby stimulating the expression of M2-related genes [214].

### Serine metabolic reprogramming induces M2-like polarization of TAMs

Serine metabolism also plays a role in the regulation of TAM polarization (Fig. 6, B, Table 3). Serine metabolism is involved in TAM polarization in response to various environmental cues [[215], [216], [217], [218]]. In the serine biosynthesis pathway, phosphoglycerate dehydrogenase (PHGDH) catalyzes the first rate-limiting step in the conversion of glycolytic-derived 3-phosphoglycerate. Exogenous serine deprivation reduces macrophage IL-1β. Interestingly, serine deprivation significantly downregulates serine hydroxymethyl transferase (SHMT1/2) expression and reduces intracellular glycine levels [216,218,219]. Moreover, the mutual conversion of serine and glycine can then be achieved by SHMT1/2, in some cancers, genomic alterations of the genes encoding the enzymes of the serine/glycine synthesis pathway result in the overexpression of SHMT1/2. Serine metabolism plays a key role in the coordination of TAM polarization [[220], [221], [222]]. The overexpression of PHGDH in TAMs in a mouse model of mesothelioma significantly reduced the expression of M1-like markers in RAW264.7 cells [223], and *in vivo* experiments revealed that myeloid PHGDH did not strongly inhibit tumor growth, suggesting that the enzyme activity of PHGDH inhibits the M2-like polarization of TAMs [224]. PHGDH in human breast tumor cells can promote the activation and proliferation of M2-like TAMs through the secretion of insulin-like growth factor 1 receptor (IGF-1) [224]. Insufficient serine metabolism increases IGF-1 expression by reducing the abundance of S-adenosylmethionine (SAM)-dependent histone H3 lysine-27 trimethylation (H3K27me3) on the promoter. IGF-1 then activates the P38-dependent JAK‒STAT1 axis, promoting the M1-like polarization of TAMs and inhibiting STAT6-mediated M2-like TAM activation [224]. Both the typical Th2 immune response and the TME enhance PHGDH-mediated de novo serine synthesis in TAMs and promote immunosuppressant TAM activation and proliferation through mTORC1 signaling [223]. The deletion of PHGDH disrupts cell metabolism and mitochondrial respiration, reduces PD-L1 production in TAMs [223], and partially restores antitumor T-cell immunity. The inhibition of PHGDH can promote the M1-like TAM state, which depends on the presence of exogenous serine and glycine. The activity of PHGDH can enhance the M2-like polarization of TAMs, a process that is not affected by exogenous serine or glycine. PHGDH-mediated serine biosynthesis promotes α-KG production, and the activation of mTORC1 signaling is conducive to maintaining M2-like TAMs in the TME [223]. Moreover, the PHGDH-mediated SSP plays a crucial role in regulating the conversion of glutamate to α-KG, promoting the activation and proliferation of M2-like TAMs [224]. Gene ablation of PHGDH in tumor-bearing mouse TAMs inhibited tumor growth, transformed M2-like TAMs to M1-like TAMs, downregulated PD-L1 expression and enhanced antitumor T-cell immunity [223]. PHGDH depletion in TAMs can inhibit tumor proliferation and enhance T-cell immunity. Inhibiting the activity of PHGDH, a key enzyme in the serine synthesis pathway (SSS), or inhibiting serine metabolism by restricting the uptake of exogenous serine and glycine can increase M1-like polarization of TAMs and inhibit M2-like polarization of TAMs both *in vitro* and *in vivo*.

### Branched-chain amino acid metabolic reprogramming inhibits the M1-like polarization of TAMs

**Fig. 7:**
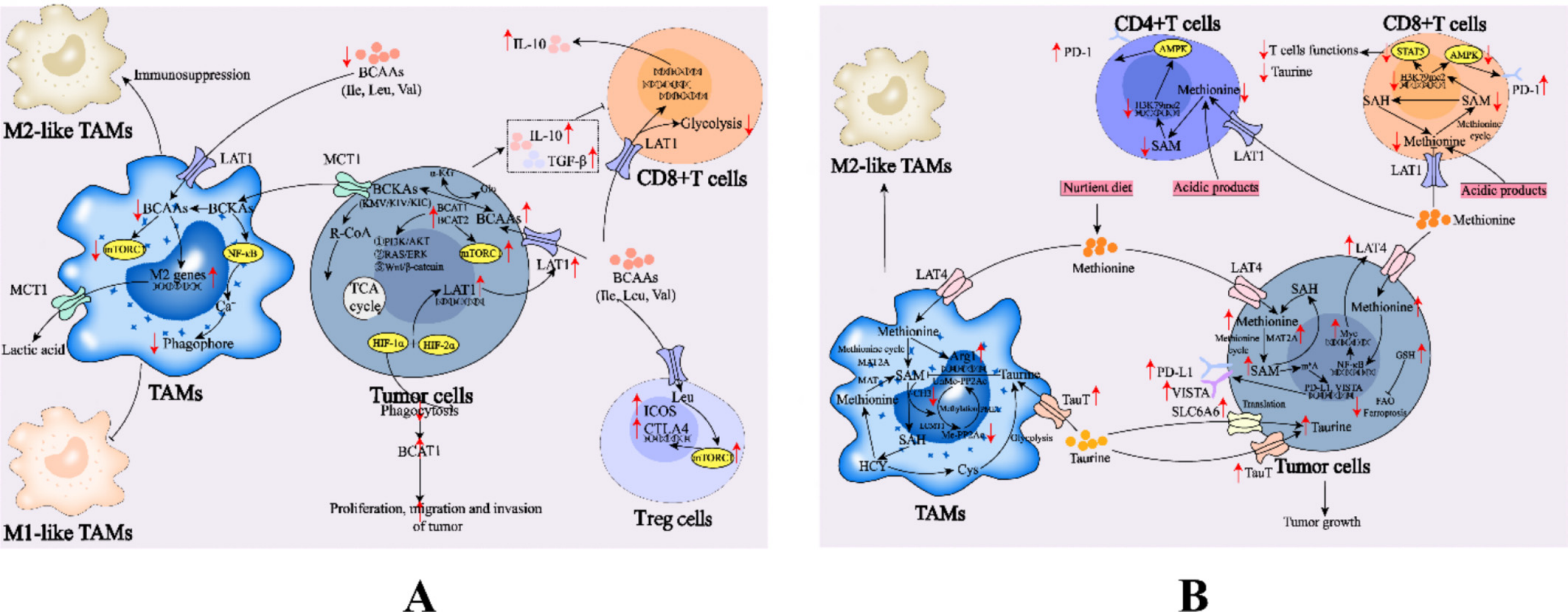
A. Role of BCAA metabolic reprogramming in TAMs. B. Role of methionine and taurine metabolic reprogramming in TAMs. *BCAAs*, branched-chain amino acids; *BCAKs*, branched-chain ketoacids; *BCAT1/2*, branched-chain amino acid transaminase 1/2; *CTLA4*, cytotoxic T-lymphocyte associated protein 4; *Cys*, cysteine; *GSH*, glutathione; *HCY*, homocysteine; *H3K79me*, methylation of lysine 27 on histone 3; *ICOS*, inducible T-cell costimulatory; *IL-1β*, interleukin-1β; *KIC*, α-ketoisocaproate; *KIV*, α-ketoisovalerate; *KMV*, α-keto-β-methylvalerate; *LCMT1*, leucine carboxyl methyltransferase 1; *MAT*, methionine adenosyltransferase; *MAT2A*, methionine adenosyltransferase 2A; *Myc*, myelocytomatosis; *PP2Ac*, protein phosphatase 2A catalytic subunit; *PME1*, phosphatase methylesterase 1; *TauT*, taurine transporter; *SAH*, S-adenosylhomocysteine; *SLC6A6*, solute carrier family 6 member 6; *STAT5*, signal transducer and activator of transcription 5.

Branched-chain amino acids (BCAAs), including leucine, isoleucine and valine, are essential amino acids for human nutrition [225]. In addition to directly participating in protein synthesis, BCAAs can also produce intermediate metabolites such as glutamate through catabolism and are associated with other metabolic pathways, glycolipid metabolism, apoptosis and autophagy [226,227]. The polarization of TAMs can be influenced by the intake of BCAA transformation products by tumor cells (Table 3). LAT1 widely exists on the tumor cell membrane and is the main pathway for BCAA uptake by tumor cells. HIF-1α and HIF-2α bind to the hypoxic response element (HRE) of the LAT1 gene promoter, synergistically upregulate LAT1 protein expression, and promote BCAA uptake by GBM cells [228]. BCAAs are metabolically transformed by branched-chain amino acid transaminase (BCAT) 1 and BCAT2 into BCKAs, and the final metabolites enter the TCA cycle. The inhibition of BCAT1 reduces itaconate production in macrophages and limits flux through the TCA cycle [229,230]. The regulatory mechanisms and physiological functions of BCAT1 and BCAT2 are significantly different. BCAT2 is overexpressed mainly in c-Myc-induced PDAC, whereas BCAT1 is overexpressed in most tumors [231,232]. High BCAT expression can promote the growth and proliferation of tumor cells but also inhibits the killing effect of CD8+ T cells on tumors [233]. In tumor cells with high BCAT expression, immunosuppressive cytokines such as IL-10 and TGF-β can be produced, thereby inhibiting the antitumor immune response [234]. In addition to key enzymes in BCAA metabolism, BCAT transfers amino groups from BCAAs to α-KG to produce their respective branched-chain ketoacids (BCKAs) and glutamate metabolic breakdown products, which similarly affect the immune response (Fig. 7, A). When BCKAs were added to the macrophage culture medium, the ability of macrophages to phagocytose granules decreased in a dose-dependent manner, suggesting that BCKAs can inhibit the immune response by influencing intracellular pathways such as the NF-κB pathway to regulate macrophage phagocytosis. After the uptake of BCKAs, the intracellular Ca^2+^ concentration and fluidity of the macrophage membrane change, affecting the formation and phagocytosis of phagosomes [235]. Studies have shown that glioblastoma cells can secrete BCKAs into the TME through MCT1 and that these BCKAs are absorbed by TAMs and reconstituted into BCAAs, whereas excessive exposure to BCKAs reduces the phagocytic activity of TAMs, suggesting that the secretion of BCKAs by tumors may inhibit tumor immunity [235]. In addition, leucine can promote mTORC1 activity in Treg cells and drive inducible T-cell costimulatory (ICOS) and cytotoxic T-lymphocyte associated protein 4 (CTLA4) expression [236].

### Methionine metabolic reprogramming inhibits the M1-like polarization of TAMs

Methionine metabolism induces M1-like TAM activation, plays a proinflammatory role characterized by TNF-α release, and regulates extracellular nucleotide metabolism. This promotes an increase in ATPase/ADPase activity in macrophages [237]. TAMs can increase methionine circulation by increasing methionine adenyltransferase Iα (MAT2A) levels [238], and methionine deficiency in the TME inhibits the M1-like polarization of TAMs [219,239]. Studies have shown that methionine can decrease Arg1 activity in TAMs and significantly increase the secretion of TNF-α, suggesting that methionine can promote the polarization of TAMs into more onco-suppressing M1-like TAMs [240,241]. The demand for methionine in LPS-induced macrophages is derived from exogenous uptake; methionine can be used as the main methyl donor for the formation and methylation of S-adenosylmethionine (SAM), and methionine deficiency can inhibit the expression of IL-1β in LPS-induced macrophages. The above studies demonstrate that methionine is involved in the formation of the M1-like TAMs. Tumor cells compete for methionine in the TME to inhibit the M1-like polarization of TAMs, further affecting the antitumor effect of TAMs [242]. Unlike normal cells, tumor cells are strongly dependent on exogenous methionine due to their lack of endogenous methionine biosynthesis, which is known as the Hoffmann effect. Tumor cells compete with T cells for methionine through large neutral AA transporter 4 (LAT4), and methionine restriction (MR) inhibits T-cell infiltration and function [243,244]. The intake of methionine by tumor cells can activate the NF-κB signaling pathway, increase LAT4 expression during tumor progression [245], and further increase methionine in the TME. Owing to the competitive consumption of TAMs by tumor cells, the decrease in exogenous methionine in the available TME inhibits their polarization to M1-like TAMs. One study revealed that the expression of M2-like-related genes significantly decreased (*Mgl2*, *Ym1*, *Relma*, and *Arg1*) after methionine starvation treatment for 48 h, whereas the expression of CD36 and FABP4 was not affected [238]. The inhibition of methionine metabolism or restriction of extracellular methionine can promote tumor cell death. Increased methionine uptake by tumor cells through LAT4 during tumor progression can outpace competition for methionine by CD8+ T cells, resulting in impaired T-cell survival and function [243]. SAM-mediated m6A methylation of PD-L1 and VISTA in tumor cells also inhibits T-cell immunity [244]. The reduction in H3K79me2 caused by SAM restriction downregulates AMPK expression in CD4+ T cells, upregulates PD-1 expression and affects antitumor immunity. Gene ablation of LAT4 in tumor cells can restore methionine metabolism in CD4+ T cells, increase the intracellular SAM level and produce H3K79me2 [246,247]. These studies suggest that elevated methionine metabolism in tumor cells may diminish the response of tumors to antitumor immunotherapy (Fig. 7, B).

### Taurine metabolic reprogramming promotes the M2-like polarization of TAMs

Taurine is the most abundant free amino acid in mononuclear macrophages, accounting for approximately 50 % of the free amino acids in cells. Since macrophages cannot synthesize taurine themselves, taurine is taken up mainly from the environment by taurine transporters on the surface of macrophages [[248], [249], [250]]. Numerous studies have shown that taurine can balance the polarization of macrophages, reduce the M1/M2 ratio, and inhibit the polarization of M1 macrophages induced by LPS and IFN-γ through metabolic reprogramming mediated by the SAM/PP2Ac methylation/mitochondrial autophagy axis [251], and the expression of markers of M2-like TAMs, such as CD206, Arg1 and IL-10 is even upregulated (Fig. 7, B, Table 3). Moreover, taurine can induce M2-like polarization (CD206+, Ym1+, Mgl1+ and Arg1+) in bone marrow-derived macrophages (BMDMs), and the level of IL-10 secreted by BMDMs is also significantly increased [252]. Taurine blocks PTEN-induced putative kinase 1 (PINK1)-mediated mitochondrial autophagy by inhibiting SAM-dependent protein phosphatase 2A catalytic subunit (PP2Ac) methylation, thus blocking the required glycolytic transformation of the energy metabolic pathway to M1. The expression of the M1 markers, cyclooxygenase-2 (COX-2), TNF-α, IL-6 and CXCL-10 is decreased, whereas the expression of the M2 markers, CD206 and IL-10 is increased [251]. The regulatory effects of taurine on macrophages have been studied mainly through the following pathways. Taurine inhibits the NF-κB signal transduction pathway in macrophages, reduces the activation of Kupffer cells in the liver, and decreases the levels of NF-κB and its downstream inflammatory factors TNF-α, IL-1β, and IL-6253 [254]. Within the TME, taurine can induce TAM-tumor cell crosstalk in an IL-6/JAK2/STAT3 axis-dependent manner during resistance to iron death due to high levels of taurine transporter (TauT) expression for taurine transport [255]. The upregulation of TauT has been observed in both M2-like TAMs and PCa cells, and TauT is also expressed in other cell types, such as CD8+ T cells [256]. TAMs output taurine to inhibit iron death by activating the LXRα/SCD1 axis in PCa [255,257]. Moreover, LXRα promotes the expression and recruitment of miR-181a-5p into EVs released by tumor cells. In response, EV-mediated miR-181a-5p shuttles into TAMs and increases the expression of the M2-like TAM polarimetric genes Arg1, CD163 and TauT by inhibiting the Hippo pathway, thereby increasing taurine secretion.

Cysteine is synthesized from methionine via the transsulfuration pathway. Another supply route involves the transport of cystine by solute carrier family 7 member 11 (xCT/SLC7A11), a transmembrane sodium-independent and chloride-dependent antiporter of cystine and glutamate [258,259]. *In vitro*, a study proposed a ‘sulfur negative feedback loop’ that regulates the inflammatory response of macrophages: sulfur metabolism supported by xCT is another key axis for regulating macrophage behavior to limit the expression of excessive proinflammatory genes. After LPS treatment, the uptake of cystine by macrophages through xCT suddenly increases, followed by an increase in the production of supersulfides; otherwise, it hinders the regression of the inflammatory response of macrophages [260]. Although previous studies reported that xCT-deficient peritoneal macrophages (xCT-KO PMs) exhibit impaired nitric oxide production and decreased viability after long-term culture [261,262], suggesting the substantial requirement of cysteine for macrophage function, how sulfur metabolites, including supersulfides, impact macrophage behavior remains to be elucidated. Cysteine, taurine and hypotaurine are typically used for M2-like polarization and are also affected by taurine metabolism, while cysteine metabolism is especially associated with the polarization of M2b, M2c and M2d [73].

## Targeting therapeutic strategies to tune amino acid metabolism in TAMs

### Depriving amino acids in the TME

Competitive amino acid metabolism in the TME by tumor cells can hinder the proliferation and polarization of immune cells, affect the availability of amino acids in TMAs, and directly supplement or deprive TAMs, which results in increased effectiveness of immunotherapy. The proliferation and differentiation of TAMs are highly dependent on amino acid metabolism, and the direct depletion of amino acid levels in the TME can cause the repolarization of TAMs in an antitumor direction, repress the immunosuppressive microenvironment, and affect tumor progression. Targeted therapies that interfere with TAM amino acid metabolic reprogramming are currently being explored in multiple clinical trials across a range of tumor types.

#### Tryptophan

Tryptophan plays an important role in promoting the M2-like polarization of TAMs and tumor progression, and the deprivation of tryptophan in the TME can inhibit the formation of an immunosuppressive microenvironment. In animal models, the removal of tryptophan from the diet effectively inhibited tumor growth, and supplementation with tryptophan or its catabolic indole-3-acetic acid increased tumor growth and promoted an immunosuppressive TAM phenotype. Studies have shown that TAMs within PDAC cells exhibit high AhR activity and that the direct restriction of tryptophan in the diet reduces AhR activity within TAMs and promotes antitumor immunity [155]. In addition, eliminating Kyn or other immunosuppressive metabolites in the TME may be an effective way to inhibit tumor development. AhR activation in TAMs may not only depend on tryptophan metabolism but also require dietary tryptophan indoles metabolized by lactic acid bacteria to drive TAMs to acquire an immunosuppressive phenotype [166]. As a Kyn sensor, AhR plays a key role in the Trp-IDO/TDO-Kyn-AhR pathway; therefore, it is often considered a therapeutic target in immune cells and tumor cells. This immunosuppressive axis may be an effective target for selectively blocking AhR to delay the progression of IDO/TDO-overexpressing tumors and enhance antitumor efficacy when used in combination with immune checkpoint blockade [266]. Ilantimod (NCT04999202 and NCT04069026), KYN-101 [166], and KYN-175 all inhibit the Trp-Kyn-AHR pathway by blocking AhR, improving immunosuppression in the TME. A phase I (NCT04200963) clinical trial involving a multicenter, open label study designed to assess the safety and tolerability of KYN-175 as a single agent and in combination with nivolumab to determine the recommended phase II dose (RP2D), indicated that KYN-175 was well tolerated and demonstrated durable antitumor activity both as a monotherapy and in combination therapy with nivolumab in patients with urothelial carcinoma (Table 4.1). PEG-KYNase is a Kyn modulator that degrades Kyn into nonimmunosuppressive metabolites and mediates Kyn accumulation caused by the increased expression of IDO1/TDO in the depleted TME [267], inhibiting the M2-like polarization of TAMs and increasing the infiltration of CD8+ T cells, thus inhibiting tumor growth. In addition, tryptophan deprivation can be combined with IDO1/TDO inhibitors to enhance efficacy [268]. 3′,4′-Dimethoxy-flavone (DMF) is a potent AhR inhibitor that blocks the formation of the nuclear AhR complex in TCDD-induced breast cancer cells [269]. DMF significantly enhances PD-1 blockade in CD8+ T cells when combined with carboxyaminotriazole [270]. The AhR inhibitor AhRil (CH-223191) promotes the M1-like polarization of TAMs and increases CD8+ T-cell infiltration, thereby improving immune checkpoint blockade therapy with PD-1 antibodies [166,271,272]. A decrease in tryptophan levels in the TME can directly inhibit the M2-like polarization of TAMs.

**Table 4.1:** Clinical trials and preclinical studies of amino acid metabolic reprogramming in TAM-targeted therapy (targeting amino acid transporters and others).

No.	Target	Agent	Mechanism	Tumor types	Trial phase	State	Combination partners	ID.
1	SLC6A8	RGX-202	Targets SLC6A8 inhibits creatine use by cells	①Colorectal cancer ②Metastatic colon cancer ③Gastrointestinal cancer ④Pancreatic cancer	I/II	Underway	5-Fluorouracil/ Leflunomide/ Bevacizumab	NCT05983367 NCT03597581
2	ASCT2	GPNA	Inhibits ASCT2, depletes Glu in the TME, decreases IL-23 secretion	Clear cell renal cell carcinoma (glutamine addiction)	Preclinical study	/	PD-1 inhibitors	[183,348]
3	EAAT	D-Asp	Inhibits EAAT2 and EAAT1, increases Glu-induced GSH in macrophages	*In vitro* experiment	Preclinical study	/	/	[266]
4	LAT1	BCH	Inhibits LAT1 and mTORC1 signaling	①Glioma ②Oral epidermoid carcinoma ③Osteogenic sarcoma	Preclinical study	/	/	[298,299]
5		JPH203	Selectively inhibits LAT1	①Advanced biliary tract cancer ②Colorectal cancer ③Renal cell carcinoma	I/II	Completed	/	JPRN-UMIN000034080 JPRN-UMIN000016840
6	mTOR	Ridaforolimus	Influences amino acids, glucose, nucleotide, fatty acids, and lipid metabolism	①Hematologic malignancies ②Metastatic endometrial cancer ③Sarcoma	III/Done	Completed	/	NCT00086125 NCT00122343 NCT00538239
7	Glutamine	Sirpiglenastat	Glutamine antagonist	Fibrolamellar hepatocellular carcinoma	I/II	Recruiting	/	NCT06027086
8	AhR	Ilantimod	Blocks the Trp-Kyn-AHR pathway	Advanced solid tumors	I	Terminated Completed	Pembrolizumab/	NCT04999202 NCT04069026
9		KYN-101		①Melanoma ②Colorectal cancer	Preclinical study	/	PD-1 inhibitors	[166]
10		KYN-175		①Advanced solid tumors ②Metastatic solid tumors ③Urothelial carcinoma ④Head and neck squamous cell carcinoma	I	Completed Withdrawn	Nivolumab	NCT04200963 NCT05472506
11		CH-223191	Targets the AhR in TAMs, alleviates T-cell dysfunction	①Pancreatic ductal adenocarcinoma ②Glioblastoma	Preclinical study	/	/	[172,349]
12	Kyn	PEG-KYNase	Kynurenine depletion, reverses upregulated IDO1/TDO expression in the TME	①Melanoma ②Breast cancer ③Colorectal cancer	Preclinical study	/	/	[267]

#### Arginine

Arginine depletion within the TME is considered a therapeutic approach for arginine dystrophic tumors (Table 4.2, Table 4.3). Arginine depletion is a clinically safe and achievable strategy in children with cancer. A global cohort of 49 pediatric patients was used to determine the recommended Phase II dose for children to be 1,600 U/kg per week (NCT03455140), matching the adult dose. These results indicated that BCT-100 had good tolerance. ADI-PEG20 is a representative arginine-depleting agent [273,274] that can convert extracellular arginine into citrate, hinder the external supply of arginine to cancer cells, and inhibit the utilization of arginine by M2-like TAMs. In breast cancer, supplementation with sepiapterin, a precursor of tetrahydrobiopterin, acts as an NOS cofactor to redirect arginine metabolism in macrophages from the polyamine synthesis pathway to the NO pathway; this action induces the metastasis of M2-like TAMs to M1-like TAMs and blocks the STAT3-dependent expression of PD-L1 in tumor cells to inhibit their growth [275]. Studies have shown that ADI-PEG20 combined with docetaxel is effective for treating lung cancer, head and neck squamous cell carcinoma, prostate cancer and other tumors. Arginine supplements have also shown efficacy when used in combination with chemotherapy agents, such as in a mouse model of breast cancer, where arginine in combination with docetaxel (DTX) promoted the antitumor phenotype of DCs and reduced the proliferation of MDSCs [276]. ADI-PEG20 an arginine deiminase enzyme is currently undergoing phase I to III clinical trials for many cancers. Although early studies of ADI-PEG20 as a single therapy were negative because of rapid metabolic adaptation, recent studies combining ADI-PEG20 with other therapies have shown positive results [273,274,277]. Two classic mechanisms of ADI-PEG20 failure involve cancer re-expressing ASS1 in a Myc-dependent manner, or by the formation of anti-ADI-PEG20 anti-drug antibodies [[278], [279], [280]]. Arg1/2 inhibitors or arginine supplementation can restore T-cell functions in different tumor models. TAMs can synthesize proline-rich collagen, consume environmental arginine and secrete ornithine, which compromises CD8+ T-cell function. Daily administration of ornithine impaired the immune control of breast tumor in a genetically engineered mouse model, whereas arginine administration improved tumor growth control [211]. These findings suggest that Arg activity inhibits T-cell function through a dual mechanism: by depleting the essential amino acid l-arginine and by producing the immunosuppressive product ornithine. However, in tumors that produce ammonia as a result of the use of amino acids as a carbon source in a glucose-deprived TME, such as breast and colon cancers [[281], [282], [283]], ornithine can enhance T-cell function by decreasing levels of ammonia, which otherwise drives T-cell exhaustion; indeed, ornithine is a substrate for glutamine synthesis, which detoxifies ammonia [284].

**Table 4.2:** Clinical trials and preclinical studies of amino acid metabolic reprogramming in TAM-targeted therapy (targeting amino acid metabolic enzymes- preclinical strategies).

No.	Target	Agent	Mechanism	Tumor types	Trial phase	State	Combination partners	ID.
1	TDO	680C91	Inhibits the AKT/GSK3β axis downregulates IL-8	①Melanoma ②Esophageal squamous cell carcinoma	Preclinical study	/	Epacadostat	[173]
2	Arg1	OATD-02 hydrochloride	Inhibits Arg1 and Arg2 and can effectively block the activity of intracellular arginase	Melanoma	Preclinical study	/	/	[336]
3	GLS1	BPTES	Inhibits GSL; decreases glutamate levels	Hepatocellular carcinoma	Preclinical study	/	/	[349]
4	GLS1/2	JHU-083	Inhibits GSL; depletes Glu in the TME	①Colon cancer ②Lymphoma ③Breast cancer ④Melanoma	Preclinical study	/	PD-1 inhibitors	[330]
5	GS	Methionine Sulfoximine	Inhibits GS; decreases the glutamine level; activates HIF-1α	Non-small cell lung cancer	Preclinical study	/	/	[178]
6		Glufosinate		Metastatic lung, skin and breast cancer		/	/	[327]
7	PHGDH	NCT-503	To investigate the relationship between pyruvate kinase M2 and serine synthesis in NSCLC	①Non-small cell lung cancer ②Colorectal cancer	Preclinical study	/	PKM2-IN-1	[345]
8		CBR-5884	Inhibits PHGDH	①Breast cancer ②Epithelial ovarian cancer	Preclinical study	/	/	[350]
9	PERK	GSK2656157	Inhibition of serine biosynthesis and immunosuppressive activity	Melanoma	Preclinical study	/	/	[351]
10	MAT2A	FIDAS-5	Competes with SAM for MAT2A binding in tumors and TAMs	①Gastric cancer ②Lung cancer	Preclinical study	/	Cisplatin	[238]

**Table 4.3:** Clinical trials and preclinical studies of amino acid metabolic reprogramming in TAM-targeted therapy (targeting amino acid metabolic enzymes- clinical strategies).

No.	Target	Agent	Mechanism	Tumor types	Trial phase	State	Combination partners	ID.
1	IO1	Epacadostat	Inhibits tryptophan metabolism; reduces kynurenine	①Muscle-invasive urothelial cancer of the bladder ②Endometrial cancer ③Renal cell carcinoma ④Urothelial cancer	I/II/III	Completed/ Enrolling by invitation/ /Active and not recuriting/ Completed/ Active and not recuriting/ Active and not recuriting	PD-1 inhibitors/ Against/ CTLA 4 antibodies	NCT03374488 NCT03328026 NCT04463771 NCT03006302 NCT03414229 NCT03358472
2		1-L-MT	Binds to the active site of IDO and prevents the accumulation of Kyn	①Neuroendocrine tumors ②Human brain tumors	II	Terminated/ Completed/ Completed	/	NCT04397679 NCT03453489 NCT02367469
3		Indoximod	Inhibits IDO1 activity, a Trp substitute	①Melanoma ②Metastatic prostate cancer ③Pancreatic cancer ④Acute myeloid leukemia	I	2025.7. /Recruiting/ Completed	PD-1 inhibitors/ Cancer vaccine	NCT05106296 NCT04049669 NCT03852446
4		BMS-986205	Specific targeting and binding to IDO1; reduces Kyn levels	①Bladder cancer ②Cervical cancer	II	Recruiting	Nivolumab	NCT02658890
5		IDO1 peptide vaccines (IDO-derived A2 peptide)	Triggers specific CD8+ T-cells; kills tumor cells and DCs expressing IDO1	Melanoma	II	Completed	PD-1 inhibitors/ Nivolumab	NCT01219348
6		CB-1158	Increases arginine metabolism via iNOS, increasing NO	Advanced solid tumors	I/II	2025.8. /Active, not recruiting/ Completed	Pembroluzimab/ PD-1 Inhibitor/ daratumumab	NCT02903914 NCT03910530 EUCTR2018-004076–35-ES
7		OAT-1746	Arg1 completely eliminated from extracellular vesicles secreted by Arg1 cells	①Advanced colorectal adenocarcinoma ②Advanced renal cell carcinoma	II	Recruiting	/	NCT05759923 CTIS2024-513006–61-00
8	iNOS	Zoledronate	Reduces IL-10, VEGF and MMP − 9 levels and restores iNOS expression	①Advanced malignant solid tumors ②Advanced prostate cancer	Approved listing	Completed/ Not yet recruiting/ Recruiting	Pembrolizumab/ ETC-159	CTRI/2023/02/050079 NCT06625190 NCT06513624
9	Arginine	BCT-100	Substitute for arginase; causes Arg depletion	①Acute myelogenous leukemia ②Advanced hepatocellular carcinoma ③Advanced solid tumors ④Lymphoma	I/II	Completed	/	EudraCT-2017–002762-44 ISRCTN-21727048 NCT03455140
10		ADI-PEG20	Converts extracellular arginine to citrulline, blocking the supply to cells	①Non-small cell lung cancer ②Pancreatic cancer ③Hepatocellular carcinoma	I/II/III	Terminated Recruiting	FOLFOX/ /Gemcitabine/ Docetaxel	NCT02102022 NCT02709512
11	GLS1/2	CB-839	Inhibits GSL; depletes Glu in the TME	①TNBC ②Non-small cell lung cancer ③Multiple myeloma ④Clear cell renal cell carcinoma (glutamine addiction)	I/II	Completed Recruiting	Paclitaxel/ Sapanisertib/ Carfilzomib and Dexamethasone	NCT03057600 NCT04250545 NCT03798678
12	MAT2A	AG-270	Inhibits MAT2A	MTAP-deleted solid tumors and lymphoma	I	Recruiting	/	NCT03435250
13		IDE-397		MTAP-deleted advanced solid tumors	I	Recruiting	/	NCT04794699
14		S095033		Advanced or metastatic esophageal squamous-cell carcinoma	I	Terminated	Paclitaxel	NCT05312372

#### Glutamine

Directly reducing the glutamine level in the TME could also impact the polarization of TAMs (Table 4.1). Glutamine deprivation inhibits UDP-GlcNAc biosynthesis and the n-glycosylation of M2-like-related proteins such as Relmα, CD206, and CD301 and restrains M2-like but not M1-like [114]. In research on human glioblastoma, the absence of exogenous glutamine significantly reduces the level of glutamate, causing the dimer GLS1 to transform into a self-assembled filamentous polymer with an extremely low km, further consuming intracellular glutamine and leading to the apoptosis of tumor cells [285]. Additionally, Rashmi *et al*. [286] found that glutamine deprivation resulted in a decrease in total reduced GSH and an increase in GSH disulfide levels in cervical cancer cells, thereby increasing intracellular oxidative stress and inducing apoptosis. Blocking glutamine metabolism does not affect T-cell function but enhances T-cell antitumor activity, and because tumor cells are addicted to glutamine, glutamine starvation has a more direct effect on tumor cell survival. Studies have shown that the addition of glutamine to the diet reverses macrophage function in newborn mice [287], and that glutamine may also inhibit lysosomal function, the anti-inflammatory phenotype, and cell survival under certain conditions [288]. Sirpiglenastat (NCT06027086) is a glutamine antagonist that can simultaneously target a variety of metabolic pathways associated with glutamine, in this trial, the primary clinical endpoints are safety and the objective response rate (ORR), defined as the percentage of patients who achieve a complete response (CR) or partial response (PR) on the basis of the response evaluation criteria for solid tumors. The secondary objectives include progression free survival (PFS), overall survival (OS), and immunological correlates, which has commenced enrollment in December 2023. Importantly, Sirpiglenastat is designed into a prodrug form, and it is activated after being cleaved by enzymes enriched in tumors, which can minimize its exposure to peripheral and gastrointestinal tissues, thereby significantly reducing toxicity [289].

#### BCAAs

Exogenous BCAA supplementation can increase the utilization of BCAAs by TAMs. Research has revealed that excess BCAAs can trigger the TLR4/NF-κB signaling pathway in macrophages [290], promoting their proinflammatory effect. Exogenous supplementation with BCAAs has synergistic effects with anti-PD-1 therapy, positioning BCAAs as complementary components to enhance the clinical efficacy of anti-PD-1 immunotherapy in cancer treatment [291]. The combination of adjusting the amino acid level in tumors with immune checkpoint inhibitors can enhance the efficacy of tumor targeting.

The key product SAM of methionine not only affects the polarization and function of TAMs, but also serves as a key product promoting tumor development. However, since methionine has extensive and important functions in normal cells, systematic deprivation of methionine to limit SAM may lead to severe side effects. Targeted intervention in methionine metabolism in tumor cells is an attractive strategy with improved efficacy and safety [247,292,293].

### Targeting amino acid transporters

Solute carrier transporters (SLCs) in TAMs are important metabolic gating agents in TAMs, and targeting SLCs can inhibit the exogenous utilization of amino acids (Table 4.1). Existing SLC inhibitors are focused mainly on tumor cells and target ASCT2, LAT1 or LAT4; additionally, some immune cells, such as Tregs, TAMs, or MDSCs, can result immunosuppression and may also be effective targets for SLC inhibitors [[294], [295], [296], [297]].

The specific LAT family inhibitor 2-aminobicyclic-(2,2,1)-heptane-2-carboxylic acid (BCH) in cancer can inhibit all members of the LAT family at concentrations higher than 10 mM, and treatment with BCH can promote the apoptosis of tumor cells such as glioma cells, oral epidermoid cancer cells, and osteosarcoma cells [298,299]. JPH203 is a selective inhibitor of LAT1, that is a high-affinity inhibitor of LAT1 with a submicromolar IC_50_ and no detectable inhibition of LAT2 [300]. A clinical trial (JPRN-UMIN000016840) has shown that the LAT1 inhibitor JPH203 shows promising activity in patients with biliary tract cancer and a high body mass index, as LAT1 subinitiation plasma free amino acids, especially BCAAs, have sufficient gradients. With such outstanding selectivity and potency, JPH203 has been proven effective against different types of cancers and successfully completed phase I and II clinical trials as a first-in-class drug against biliary tract cancer. Compared with the placebo group, the JPH203 group had significantly longer PFS as shown by blinded, independent central image assessment (JPRN-UMIN000034080) [301,302]. The current clinical trial directions of JPH203 can focus on (1) verifying the therapeutic efficacy in various types of cancer, (2) evaluating models for the lack of LAT1 distribution in normal tissues and other pathologies (especially inflammation), and (3) verifying tolerance and clinical safety. The selective inhibition of LAT1 can inhibit the presentation of tryptophan, glutamine and BCAAs in the TME to tumors or TAMs and restore the immunosuppressive microenvironment. RGX-202 is an oral inhibitor that can effectively inhibit the utilization of creatine by macrophages and promote the expression of M1-like genes [210]. A clinical trial submitted in 2023 (NCT05983367) combining FOLFIRI with bevacizumab for the treatment of advanced/metastatic colorectal cancer is ongoing. A total of 70 patients were recruited for this study, patients were randomly assigned to receive RGX-202 3000 mg BID or a matching placebo, and follow-up was expected to last at least 24 months [303].

The combination of PD98095/SB203580 can regulate the expression of solute carrier family 7 member 2 (CAT2) by inhibiting MAPK/MEK, resulting in the complete blockade of Arg transport in macrophages activated by LPS/IFN-γ; however, this process does not affect iNOS synthesis [304]. Excitatory amino acid transporter (EAAT), a transporter of glutamine on TAMs, is competitively inhibited by D-Asp *in vitro*, and the inhibition of SLC1A2 and SLC1A3 increases glutamate-induced GSH in monocyte-derived macrophages. IL-4 secreted by immune cells can increase the expression level of the glutamine transporter ASCT2 in breast cancer cells. V-9302 is a competitive antagonist of transmembrane glutamine flux and has inhibitory effects on a variety of tumor cells [296,305,306]. When V-9302 is used to treat glutamide-dependent tumors, the corresponding biomarkers need to be verified. This is because whether V-9302 responds largely depends on the activity of the ACST2 transporter rather than the expression of the ACST2 protein [305]. In some 143B osteosarcoma cells, V-9302 inhibited sodium-neutral AA transporter 2 (SNAT2) and LAT1, which are inhibitors of glutamine uptake, in HCC1806 breast cancer cells [307]. In KRAS-mutated colon cancer cells, SLC25A22-mediated glutamine breakdown reduces H3K4me3 methylation, stimulates the activation of the Wnt/beta-catenin signaling pathway, and diminishes the sensitivity of colon cancer cells to chemotherapeutic agents [308]. Given the glutamine dependence of ocitinib-resistant lung cancer cells, targeting GLS1 alone has limited antitumor effects. Clinical trials have demonstrated that a dual-targeting approach, which simultaneously inhibits ASCT2 and GLS1, may be a promising strategy for sensitizing ocitinib-resistant cells [309].

L-γ-Glutamyl-p-nitroanilide (GPNA) is a potent selective inhibitor of the glutamine transporter ASCT2, which can cause glutamine deprivation in the TME, promote the secretion of IL-23 by TAMs, and activate CD8+ T cells while polarizing TAMs to the M1-like phenotype [304,310]. BMS-986205 (NCT02658890) is an irreversible IDO1-specific targeted inhibitor. In clinical studies, BMS-986205 has been found to reverse tumor-induced immunosuppression by inhibiting IDO1 and reducing the Kyn level in tumor cells. An increase in tumor-infiltrating CD8+ T cells and a decrease in Kyn have been observed in patients with bladder and cervical cancer. In a phase II clinical trial, an IDO1-targeted peptide vaccine (IDO-derived A2 peptide, NCT01219348) was used to trigger specific CD8+ T cells to kill IDO1-expressing tumor cells and DCs and was more effective when combined with PD-1 inhibitors.

### Targeting amino acid metabolic enzymes

In addition to the abovementioned regulatory approaches for determining the concentrations of free amino acids, membrane-binding transporters, and sensors (such as mTOR and AhR) of TAMs, key metabolic enzymes also critically affect the control of TAM differentiation and function and can be used as targeted therapeutic targets (Table 4.2, Table 4.3).

#### Tryptophan metabolic enzymes

IDO1 is a tryptophan metabolic enzyme that plays an important role in the formation of an immunosuppressive TME, especially affecting the immunosuppressive effect of TAMs. The L-isomer of methylated tryptophan (1-L-MT) is a competitive inhibitor of IDO1 and, when combined with a CTLA-4 blocker, exerts a more effective antitumor immune effect in melanoma mouse models [311]. Epacadostat is a tryptophan-competitive IDO1 enzyme inhibitor [312]. The IDO peptide vaccine (NCT01219348) has good tolerance and the administration period can last up to 5 years. Among the 15 patients, 2 of them were long-term responders who continued to experience clinical reactions within 6 years after the first vaccination. *In vitro* studies have revealed that epacadostat promotes the proliferation of T cells and NK cells, increases the number of CD86+ NK cells with high DCs and decreases the number of Treg cells [313], which can suppress Kyn levels in plasma and tumors by 90 % in model mice. Studies have shown that high IDO1 expression is positively associated with microvascular density and poor prognosis in breast cancer patients [314], whereas the administration of the IDO1 inhibitor epacadostat significantly reduces neovascularization in mice with tumors or vascular metastases [315]. Epacadostat can be eliminated in immunodeficient mice, which suggests that it inhibits tumor growth by stimulating an immune response. When epacadostat was used in combination with anti-PD-1 antibodies, a significant improvement in the survival rate was observed in C57BL/6 miceh intracranially implanted with GL261 glioblastoma cells [316]. Epacadostat did not result in objective responses in advanced cancer patients when it was used as a single agent. This lack of activity as a monotherapy was also evident in a phase II clinical study in patients with myelodysplastic syndrome (MDS) treated orally with 600 mg epacadostat BID for 16 weeks, as well as in a phase II clinical trial in patients with advanced epithelial ovarian, primary peritoneal, or fallopian tube cancer [317,318]. In two open-label, phase I/II studies of patients with advanced melanoma, the combinations of epacadostat plus pembrolizumab (ECHO-202) [319] and epacadostat plus nivolumab (ECHO-204) [320] showed promising antitumor activity compared with historical data. However, in the phase III (ECHO-301/KEYNOTE-252) study, the addition of 100 mg of epacadostat twice daily to pembrolizumab did not result in greater clinical benefit than pembrolizumab monotherapy (PD-L1 inhibitor) in patients with unresectable or metastatic melanoma previously untreated with a checkpoint inhibitor. The possible reasons are that there are differences in research design among the studies, different inclusion criteria, and the importance of IDO1 expression as a target in melanoma remains controversial, because studies in the past decade in human melanoma tissue have shown notable heterogeneity in IDO expression in longitudinal samples [321].

In addition, the combination of IDO1 inhibitors with PD-1/L1 and CTLA-4 antibodies has shown significant synergistic antitumor efficacy in mouse models of brain cancer and melanoma. At the 2017 American Society of Clinical Oncology (ASCO) meeting, the latest phase I/II clinical trials was presented in the form of a series of posters. Adding epacadostat to checkpoint inhibitors seems to be well tolerated. Preliminary phase I/II clinical data indicate that when epacadostat is used in combination with pembrolizumab, non-small cell lung cancer (NSCLC) (ORR: 35 %), renal cell carcinoma (RCC) (ORR: 47 %), neck squamous cell carcinoma (SCCHN) (ORR: 34 %), and bladder cancer (ORR: overall response rate (ORR) of cancer (35 %) have improved. The FDA-approved agents ipilimumab (anti-CTLA-4 antibody), nivolumab (anti-PD-1 antibody), and pembrolizumab (anti-PD-1 antibody) have been combined with epacadostat for the treatment of these tumors [[322], [323], [324], [325]]. On the basis of the encouraging results of NSCLC, SCCHN, RCC and urothelial carcinoma, phase III trials of epacadostat and pembrolizumab are planned. Furthermore, the combination of epacadostat and nivolumab also led to an increase in the ORR for patients with SCCHN and newly treated melanoma, and the response seemed to be independent of tumor PD-1 expression. Other combined strategies include tumor vaccines, chemotherapy and molecular targeted kinase inhibitors. However, compared with the promising results obtained with epacadostat, the combination of navoximod and FFDA approved atezolizumab (anti-PD-L1 antibody) led to only partial remission in 9 % of patients and stable conditions in 24 % of patients with ≥ 1 in-treatment tumor assessment [162].

Indoximoids (NCT05106296, NCT04049669, and NCT03852446), as IDO1 inhibitors, have poor antitumor efficacy as single drugs, but when combined with other therapies, such as PD-1 checkpoint inhibitors, their efficacy is significantly enhanced, increasing the activity of T cells in tumors and improving the immunosuppressive microenvironment [326]. BMS-986205 (NCT02658890) is an irreversible IDO1-specific targeted inhibitor. In clinical studies, BMS-986205 has been found to reverse tumor-induced immunosuppression by inhibiting IDO1 and reducing the Kyn level in tumor cells. An increase in tumor-infiltrating CD8+ T cells and a decrease in Kyn have been observed in patients with bladder and cervical cancer. In a phase II clinical trial, an IDO1-targeted peptide vaccine (IDO-derived A2 peptide, NCT01219348) was used to trigger specific CD8+ T cells to kill IDO1-expressing tumor cells and DCs and was more effective when combined with PD-1 inhibitors. TDO can participate in the catabolism of tryptophan, increase Kyn levels in the TME, and affect the polarization direction of TAMs. Preclinical studies have shown that 680C91 can inhibit TDO in melanoma and esophageal squamous cell carcinoma models, that the AKT/GSK3β axis can be inhibited to downregulate IL-8 expression, and that M2-like TAMs can be re-educated into M1-like TAMs, reducing Kyn levels to restore immunity and promote the polarization of TAMs toward antitumor phenotypes [166].

#### Glutamine metabolic enzymes

In addition to tryptophan-metabolizing enzymes, glutamine deprivation has been shown to inhibit M2-like TAMs, and targeting glutamine anabolic- and catabolic-related enzymes can effectively improve the immunosuppressive TME. Methionine sulfoximine A, a highly specific and irreversible inhibitor of glutamine synthetase, inhibits GS, reduces glutamine levels, and activates HIF-1α to re-educateTAMs [327]. BPTES inhibits the consumption of GSL and glutamate in the TME and enhances the invasion of M1-like TAMs in hepatocellular carcinoma [328], and combining BPTES with anti-PD-1 immunotherapy will improve its efficacy [329]. In a phase I/II clinical trial, CB-839 (NCT03057600, NCT04250545, NCT04250545, and NCT03798678) inhibited the consumption of GSL and glutamate in the TME, transformed M2-like TAMs into M1-like TAMs, and activated CD8+ T cells. JHU-083 is an orally effective, selective glutaminase antagonist with tumor therapy potential that reduces glutamate levels in animals, the conversion of M2-like TAMs to M1-like TAMs, and the activation of CD8+ T cells [330,331]. In ovarian cancer, FA-DCNPs inhibit the production of glutamate through the folate receptor (FOLR). Targeting M2-like TAMs can reduce the M2-like polarization of TAMs and increase the number of M1-like TAMs, thereby enhancing the antitumor immune microenvironment [332]. In prostate cancer and bladder cancer, the glutamine metabolism antagonist JHU083 inhibits TAM glutamine metabolism, resulting in increased glycolysis, disruption of the TCA cycle and impaired purine metabolism. This promotes the stem cell-like phenotype of CD8+ T cells and reduces the population of Treg cells [331], thereby enhancing the antitumor immune response. Current drugs can be classified as those that directly target upstream molecules that affect glutamine synthesis or those that indirectly target some key molecules downstream to affect the role of glutamine in the development of tumor cells. However, glutamine is an important substrate of the immune system. The long-term use of glutamine metabolic inhibitors may weaken the antitumor immune response. Therefore, it is necessary to study how glutamine metabolism affects tumor cell proliferation and immune cell function to help create new metabolic therapies.

#### Arginine metabolic enzymes

Arg1 is another key factor in arginine metabolism in immunosuppressive myeloid cells and tumors. The Arg1 small molecule inhibitor CB-1158 is an effective and orally active arginase inhibitor that can inhibit a variety of advanced solid tumors by promoting arginine metabolism to produce NO through the upregulation of iNOS expression. Inducing the transformation of the TME to inhibit immune escape can effectively reduce tumor growth [212,333]. CB-1158 has high oral bioavailability in both mice and rats. Twice-daily oral dosing of CB-1158 produced dose-dependent pharmacodynamic increases in plasma and tumor arginine levels and resulted in single-agent antitumor efficacy in several murine syngeneic tumor models including LLC, Madison-109 lung carcinoma and B16F10 melanoma. Immunodepletion of either CD8+ T-cells or NK-cells partially antagonized the antitumor effect of CB-1158 in the LCC and B16F10 models indicating that CB-1158 acts via an immune cell-mediated mechanism [334]. In clinical trials (NCT02903914, NCT03910530, EUCTR2018-004076-35-ES), the combination of pembroluzimab/PD-1 inhibitor/daratumumab has also produced good therapeutic effects. OAT-1746 is another Arg1 inhibitor that completely eliminates the immunosuppressive function of Arg1 in extracellular vesicles secreted by cells [335], and M2-like polarization of TAMs can be further inhibited. OATD-02 hydrochloride is an orally effective, competitive, reversible, noncovalently bound dual inhibitor of Arg1 and Arg2 that effectively blocks intracellular arginase activity and eliminates two types of arginase-induced tumor immunosuppression in melanoma [336]. OATD-02 monotherapy has antitumor effects on multiple tumors and enhances the efficacy of other immunomodulators. Completed nonclinical studies and human pharmacokinetic predictions have indicated feasible treatment windows and allowed for the proposal of dose ranges for the first human clinical study of cancer patients [336]. Inhibitors based on arginase are being developed rapidly, and most of them are drugs that directly target arginine metabolism in all cells in the TME.

#### Serine metabolic enzymes

Serine metabolism promotes tumor proliferation by participating in many important processes, and therapies such as PHGDH inhibitor therapy or dietary restriction have been extensively studied in animal models [[337], [338], [339], [340], [341], [342], [343]]. The role of PHGDH in various cancer types offers promising options for new treatment strategies. In a recent study, the combined application of the pyruvate kinase M2 inhibitor PKM2-IN-1 and the PHGDH inhibitor CNT-503 in human NSCLC A549 cells was investigated [344], and M2-like TAMs were re-educated into M1-like TAMs. The results of mouse experiments confirmed that the effectiveness of NCT-503 therapy combined with radiotherapy increased [345]; this study confirmed that the PHGDH inhibitor CBR-5884 can be used to treat not only breast cancer but also epithelial ovarian cancer and that the immunosuppressive effect of M2-like TAMs can be suppressed by inhibiting PHGDH. CBR-5884 can directly upregulate the mRNA expression of M1-specific marker genes (including *il-6* and *il-1β*) and significantly increase the protein expression of iNOS in RAW264.7 cells and BMDMs. More research on serine metabolism within the TME, especially in tumors or immune cells, should be conducted to determine whether existing PHGDH inhibitors can be applied to various types of cancer.

#### Methionine metabolic enzymes

Methionine adenosine transferase (MAT) regulates SAM biosynthesis, which can profoundly affect the growth, differentiation and function of cells and can be divided into MAT1A, MAT2A and MAT2B. For intratumoral methionine metabolism. MAT2A is key to the vulnerability of tumors, and the inhibition of MAT2A significantly inhibits tumor growth and improves survival in DMG mice, increasing their life expectancy by nearly 50 % on a SAM-restricted diet [346]. The expression level of MAT2A mRNA in gastric cancer tissues is much greater than that in normal tissues, and MAT2A can also participate in the regulation of TAMs in gastric cancer, activating and maintaining the tumor-promoting phenotype of TAMs through the MAT2A/WDR5/RIP1 axis. FIDAS-5 can compete with SAM to bind MAT2A in tumor cells and TAMs, inhibit the gene expression of M2-like TAMs, and reeducate M2-like TAMs into M1-like TAMs [238]. Phase I clinical studies have shown that AG-270 (NCT03435250), IDE-397 (NCT04794699), and S095033 (NCT05312372) inhibitory effects on MAT2A in a variety of tumors and can effectively improve the immunosuppression caused by methionine. Cullin 3 (CUL3, a scaffold protein that assembles many ubiquitin ligase complexes) can target MAT2A and promote MAT2A proteasome degradation through a ubiquitination (Ub)-mediated pathway [347]. MAT2A can play a positive or negative role in various cancers, and targeting MAT2A may become a new cancer treatment approach; however, at present, improving the selectivity of MAT2A inhibitors for methylthioadenosine phosphorylase (MTAP) −deficient cancers remains a major challenge.

## Conclusion and future perspectives

Specific metabolic characteristics are the driving factors for the immunosuppressive effect of TAMs in the TME. The imbalance of amino acid consumption in TAMs is an important potential mechanism leading to impaired antitumor immunity, and targeted changes in the metabolic profile can repolarize TAMs and activate tumor-killing immunity. A series of amino acid transporters and rate-limiting enzymes involved in amino acid metabolic reprogramming jointly regulate the function of TAMs. In addition to tryptophan, arginine, glutamine, taurine and other transporters, direct amino acid deprivation and targeting a series of amino acid metabolic pathway rate-limiting enzymes and receptors are effective strategies. On the basis of the negative effects of metabolic imbalance on antitumor immunity, the inhibition of amino acid metabolism and the reversal of amino acid imbalance in the TME could be effective supplements in antitumor immunotherapy. At present, most studies focus on a small number of amino acids and regulatory pathways in immune cells, such as uptake processes and metabolite functions, and lack a detailed understanding of the mechanism driving the metabolic reprogramming of immune cells, especially the specific metabolic reprogramming process of amino acids for TAMs and the resulting effects. The role of other amino acids in tumor-associated immune cells, as well as their metabolic processes and functional metabolites, still requires further investigation. A full understanding of amino acid metabolic reprogramming in immune cells in the TME, especially TAMs, is conducive to the development of antitumor strategies based on metabolism‒immunity pathways.

With the identification of numerous therapeutic targets and the exploration of drug candidates to modulate TAMs (Table 4.1, Table 4.2, Table 4.3), combining amino acid metabolic reprogramming targeting TAMs within the TME with other interventions (CAR-T cells, exosomes [352], nanoparticles [353] and oncolytic viruses) has the potential to enhance antitumor immune responses more effectively or reverse resistance [[354], [355], [356], [357], [358], [359]]. Adenoviruses (ADVs) expressing Tα1 can turn M2-like TAMs into M1-like TAMs, significantly increasing the number of tumor-infiltrating T cells in the TME [360,361]. ADVs may be effective vectors for targeting amino acid metabolism in TAMs to promote their repolarization. Advances in gene editing technology can also effectively silence genes that promote the tumor-promoting function of TAMs (e.g., Arg1 and IDO1). In addition to single drugs, drug combinations are also worth discussing. Compared with monotherapy (anti-PD-1 or chemotherapy), combination therapy with amino acid supplements or metabolic enzyme inhibitors has greater efficacy. For example, IDO, IDO2 and TDO combined to ablate tryptophan catabolism products can be used as endogenous regulatory strategies for immune activation inhibitors. Taken together, these results suggest that a combined strategy targeting multiple immune checkpoints may be the weapon of choice in the future.

Despite these breakthroughs, many key issues remain to be explored regarding the reprogramming of amino acid metabolism in the TME. (1) Amino acids are essential metabolites required for many physiological processes, and systemic cytotoxicity should be avoided when TAM-specific amino acid metabolism is targeted. Switching to a new delivery system may be an effective strategy. (2) TAMs show great variation in amino acid metabolism and phenotype and function in different tumor settings; thus, further analyses of their heterogeneity and plasticity by integrating multiomics analyses at the single-cell and subcellular levels are necessary. (3) The specific metabolic enzymes and intermediates that regulate TAMs in the tumor-specific TME, as well as the spatiotemporal regulatory mechanisms of metabolic networks in TAMs, need to be further explored to better understand their mechanisms of action in complex TME structures. (4) In clinical studies, the amino acid metabolism characteristics, mode of action and targeted drugs used by TAMs still need to be further explored. The analysis of these problems will help to deepen the understanding of the tumor immune metabolic regulatory network and provide new ideas for the development of tumor therapies that target TAMs.

Funding information: The research was supported by the Key R&D Program of Shandong Province, China (202502), the Shandong Provincial Laboratory Project (SYS202202), Shandong Provincial Natural Science Foundation (ZR2025MS1306), National Natural Science Foundation of China (82272819 and 81972888), Research Project of Jinan Microecological Biomedicine Shandong Laboratory (JNL-2025008B, JNL-2025009B, JNL-2025011B, JNL-2025010B, and JNL-2023017D), and Primary Research and Development Plan of Jiangsu Province (BE2022840).

## Compliance with Ethics Requirements

Our article does not contain any studies with human or animal subjects.

## Declaration of competing interest

The authors declare that they have no known competing financial interests or personal relationships that could have appeared to influence the work reported in this paper.
